# Comparative Phytochemical Profiles and Antioxidant Potential of Green and Variegated Morphotypes of *Alternanthera bettzickiana* Under Optimized Extraction Conditions

**DOI:** 10.1002/fsn3.72237

**Published:** 2026-08-11

**Authors:** Sirinapa Thangsiri, Woorawee Inthachat, Piya Temviriyanukul, Amornrat Aursalung, Yuraporn Sahasakul, Uthaiwan Suttisansanee

**Affiliations:** ^1^ Food and Nutrition Academic and Research Cluster, Institute of Nutrition Mahidol University Phuttamonthon Nakhon Pathom Thailand

**Keywords:** food security, functional food, plant‐based bioactive compounds, response surface methodology, sustainable food source

## Abstract

*Alternanthera bettzickiana* exhibits considerable morphological diversity, which has contributed to significant inconsistencies in reported phytochemical profiles and bioactivities, as the majority of published studies have not specified the morphotype of the plant material examined. Unlike the green morphotype, which is widely utilized in culinary applications, the variegated (mixed red and green) morphotype has received considerably less attention as an edible vegetable. Furthermore, no validated extraction protocol has been established for this species, despite being a critical determinant of bioactive compound recovery. The present study therefore aimed to optimize extraction conditions and compare the phenolic profiles and antioxidant capacities of both morphotypes. Utilizing Box–Behnken design (BBD)‐based response surface methodology (RSM), the optimized extraction conditions comprised 90% (*v/v*) aqueous ethanol, a shaking temperature of 50°C, an incubating time of 2 h, and an extract concentration of 10 mg/mL. Under these conditions, the green morphotype exhibited higher total phenolic content (4.95 mg gallic acid equivalent/g dry sample weight (DW)), whereas the variegated morphotype exhibited higher total flavonoid content (31.95 mg quercetin equivalent/g DW) and total anthocyanin content (7.04 mg cyanidin‐3‐*O*‐glucoside equivalent/g DW). Liquid chromatography‐electrospray ionization‐tandem mass spectrometry identified *p*‐coumaric acid as the predominant phenolic compound, followed by 3,4‐dihydroxybenzoic acid, in both morphotypes, while rutin was exclusively detected in trace amounts in the green morphotype. The green morphotype also demonstrated significantly higher ferric reducing antioxidant power (FRAP) and oxygen radical absorbance capacity (ORAC) activities, whereas 2,2‐diphenyl−1‐picrylhydrazyl (DPPH) radical scavenging activities were not significantly different between morphotypes. These findings establish a scientific basis for the future valorization of both morphotypes as functional food ingredients and nutraceutical sources.

## Graphical abstract

Green and variegated *Alternanthera bettzickiana* morphotypes were compared after optimizing extraction (Box–Behnken design–RSM: 90% ethanol, 50°C, 2 h, 10 mg/mL). The green morphotype had higher phenolic content, FRAP, and ORAC activity, while the variegated morphotype had higher flavonoid and anthocyanin content. p‐Coumaric acid and 3,4‐dihydroxybenzoic acid dominated both profiles. DPPH activity was similar between morphotypes, supporting both as functional food candidates.

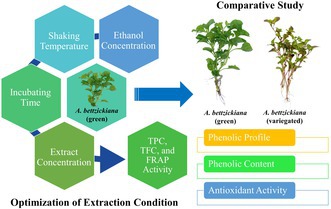

## 1 Introduction

*Alternanthera bettzickiana* is a member of the family Amaranthaceae, distributed across tropical and subtropical regions worldwide, including Thailand. The plant produces erect or creeping stems, reaching approximately 10–20 cm in height, with extensive lateral branching. The stems are slightly succulent and mechanically fragile, bearing trichomes at the shoot apices and nodes. Flowers are sessile, arising at leaf axils and branch tips. This species exhibits considerable morphological variation, particularly with respect to leaf shape, which ranges from ovate to lanceolate, and leaf pigmentation, which has led to the recognition of multiple morphotypes (Larsen 1992). Previous studies have identified three cultivars of *A. bettzickiana* distinguished by leaf coloration, comprising red, green, and variegated (mixed red and green) forms (Li et al. 2022). The two most commonly encountered morphotypes in Thailand are the green morphotype and the variegated morphotype. The green morphotype is widely utilized as a food source and incorporated into a variety of culinary preparations, including fresh salads, soups, and stir‐fried dishes. In contrast, the variegated morphotype has received considerably less attention as an edible vegetable, primarily due to its distinctive leaf pigmentation, which renders it more suitable for ornamental purposes and has consequently limited its recognition and utilization as a food source.

Previous research has demonstrated that considerable morphological diversity within *A. bettzickiana* has resulted in significant inconsistency regarding the plant material investigated across published studies, with specimens sharing the same taxonomic name potentially representing distinct botanical entities (Li et al. 2022). Consequently, only a single study has specifically characterized the green morphotype of *A. bettzickiana* , reporting total phenolic content (TPC) by spectrophotometric methods and phenolic profile identification by high performance liquid chromatography (HPLC) (Saengha et al. 2022). Both methods, however, present notable analytical limitations. Spectrophotometric TPC quantification is susceptible to interference from non‐phenolic reducing substances, which may contribute to overestimation of the true phenolic content (Dominguez‐López et al. 2024), while the HPLC approach, relying solely on spectral fingerprint and retention time comparisons against reference standards, lacks the sensitivity and specificity of contemporary techniques such as liquid chromatography coupled with electrospray ionization tandem mass spectrometry (LC–ESI–MS/MS), which enables unambiguous structural identification and precise quantification of individual phenolic compounds (Niessen 2003; Pitt 2009).

Phenolic compounds are plant‐derived secondary metabolites that act as potent dietary antioxidants, countering oxidative stress and chronic inflammation and thereby helping to protect against conditions such as cardiovascular disease, type 2 diabetes, and certain cancers (Rahman, Rahaman, et al. 2021; Sun and Shahrajabian 2023). However, only a single study has previously reported the antioxidant capacity of *A. bettzickiana* (green morphotype), employing the ferric reducing antioxidant power (FRAP) and the 2,2‐diphenyl−1‐picrylhydrazyl (DPPH) radical scavenging assays (Saengha et al. 2022). Antioxidant activity is generally evaluated through two principal mechanistic approaches, single electron transfer (SET) and hydrogen atom transfer (HAT), which reflect distinct aspects of antioxidant behavior, and the concurrent employment of both is recommended for a comprehensive evaluation of antioxidant potential (Huang et al. 2005). SET‐based methods, such as the FRAP and DPPH radical scavenging assays, measure the ability of an antioxidant to donate an electron to reduce a free radical or oxidant, whereas HAT‐based methods assess the capacity of an antioxidant to quench free radicals by donating an electron and hydrogen atom (Munteanu and Apetrei 2021). However, both assays employed in the aforementioned study operate exclusively through a SET mechanism, with no HAT‐based assessment incorporated, rendering the existing characterization of the antioxidant potential of this species incomplete.

In addition to analytical methodology, the extraction process represents a critical determinant of the yield and composition of bioactive compounds recovered from plant matrices, with extraction efficiency influenced by multiple parameters including solvent type and concentration, extraction temperature, duration, and sample‐to‐solvent ratio (Sun et al. 2025; Weremfo et al. 2023). Suboptimal extraction conditions may result in incomplete compound recovery, leading to underestimation of the true bioactive potential of the plant material. Despite the recognized importance of extraction optimization, no validated extraction protocol has been established for *A. bettzickiana* , representing a significant gap in the existing literature that necessitates systematic investigation to maximize phenolic compound recovery and ensure the reliability of subsequent biological activity assessments.

The present study therefore aimed to investigate and optimize the extraction conditions of *A. bettzickiana* to maximize phenolic compound recovery and antioxidant activity. Response surface methodology (RSM) employing a Box–Behnken design (BBD) was employed as a systematic statistical approach for the simultaneous optimization of key extraction parameters, including ethanol concentration, shaking temperature, incubation time, and extract concentration. Conventional extraction methodology was deliberately selected over advanced extraction technologies, with the primary objective of facilitating scalability and practical applicability within the industrial sector. Following the determination of optimal extraction conditions, the phenolic composition and content, alongside the antioxidant capacity, of the green and variegated morphotypes of *A. bettzickiana* were systematically compared. The phenolic profile was comprehensively characterized using LC–ESI–MS/MS, alongside spectrophotometric quantification of TPC, total flavonoid content (TFC), and total anthocyanin content (TAC). Antioxidant capacity was subsequently assessed through both SET‐based and HAT‐based mechanistic approaches to ensure a thorough evaluation of the antioxidant potential of the optimized extract. The findings of this study provide a scientifically robust foundation for the valorization of both the green and variegated morphotypes of *A. bettzickiana* as functional food ingredients and potential nutraceutical sources. Furthermore, the optimized conventional extraction protocol established herein offers practical guidance for the large‐scale production of bioactive‐rich extracts, supporting the future development of value‐added products within the food and health industries.

## 2 Materials and Methods

### 2.1 Sample Collection, Preparation, and Extraction

**FIGURE 1:**
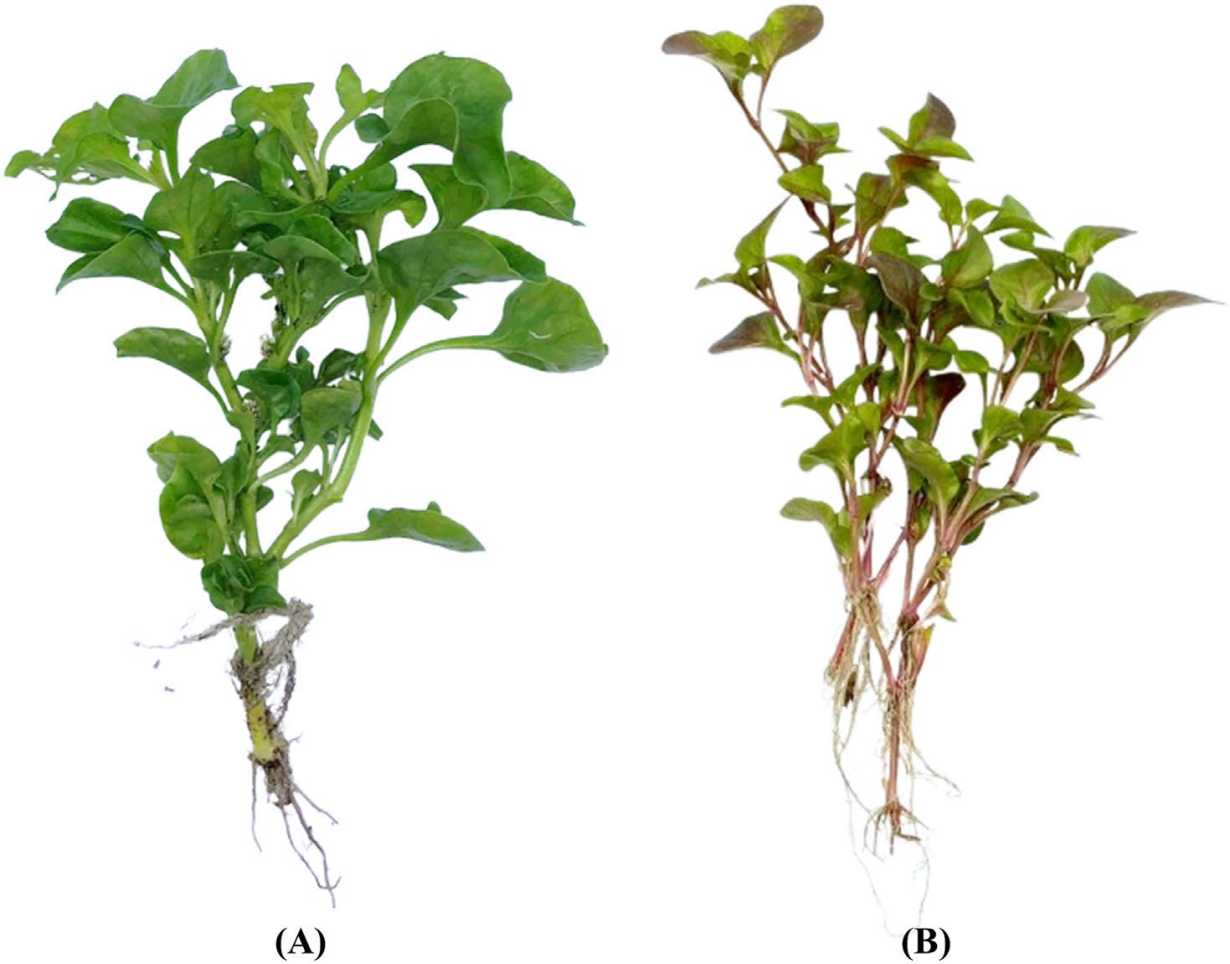
Whole plants of (A) *Alternanthera bettzickiana* (green morphotype) and (B) *Alternanthera bettzickiana* (variegated morphotype).

Young leaves of *Alternanthera bettzickiana* (5–7 cm from the shoot apex; 8–10 leaves per sample) representing the green and variegated morphotypes were collected from Nakhon Pathom Subdistrict, Mueang Nakhon Pathom District, Nakhon Pathom Province, Thailand (13°50′19.1″N, 100°03′29.0″E) in March 2022 (Figure 1). Voucher specimens were deposited at the Sireeruckhachati Nature Learning Park Herbarium, Mahidol University (Nakhon Pathom, Thailand), and assigned accession numbers PBM005783 and PBM005782 for the green and variegated morphotypes, respectively. Botanical identification and authentication were conducted by Dr. Sunisa Sangvirotjanapat (Mahidol University, Nakhon Pathom, Thailand), who confirmed both morphotypes as *Alternanthera bettzickiana* (Regel) G. Nicholson in accordance with an established taxonomic reference (Larsen 1992).

Fresh young leaves of *A. bettzickiana* were rinsed with deionized water, air‐dried for 3 h, and subsequently freeze‐dried using a −50°C and 0.086 mbar PL9000 freeze dryer (Heto Lab Equipment, Allerød, Denmark) for 72 h. The dried leaves were then pulverized using a Philips 600 W grinder (Philips Electronics Co. Ltd., Jakarta, Indonesia), and the resulting powder was passed through a 40‐mesh sieve, as verified using a Retsch AS200 sieve shaker (Retsch GmbH, Haan, Germany). The colorimetric properties of both fresh and powdered samples were evaluated using a ColorFlex EZ spectrophotometer (Hunter Associates Laboratory, Reston, VA, USA). Moisture contents of the dried samples were subsequently measured using a Halogen HE53 moisture analyzer (Mettler‐Toledo AG, Greifensee, Switzerland). The results of both analyses are presented in Table S1. For long‐term preservation, the powdered samples were sealed in aluminum foil bags and stored at −20°C until required for further analysis.

### 2.2 Optimization of Extraction Conditions

The optimization of extraction condition was performed using *A. bettzickiana* (green) as a model system, owing to the greater availability of raw material relative to *A. bettzickiana* (variegated). The standard extraction procedure was performed according to previous literature (On‐Nom et al. 2024), which involved shaking powdered samples in a temperature‐controlled water bath shaker (Memmert GmbH, Schwabach, Germany) for a designated period. It should be noted that the extraction solvent (ethanol concentration), shaking temperature, incubation time, and extract concentration were deliberately varied as independent variables throughout the optimization process, and therefore no fixed values are specified here; the specific parameter ranges and levels investigated are detailed in Sections 2.2.1 and 2.2.2, respectively. The extract concentration as used throughout this manuscript refers specifically to the solid‐to‐liquid ratio employed during the extraction procedure, expressed as the mass of freeze‐dried plant powder (mg) dissolved or suspended per unit volume of extraction solvent (mL), and therefore carries units of mg/mL. The resulting mixture was then centrifuged at 3800 × *g* for 10 min using a Hettich ROTINA 38R centrifuge (Andreas Hettich GmbH, Tuttlingen, Germany) to collect the supernatant, which was subsequently filtered through a 0.22 μm polyethersulfone (PES) syringe filter. The extract was stored at −20°C until further analysis. The optimized conditions were subsequently applied to both morphotypes for phenolic compound identification, quantitative analysis, and antioxidant capacity assessment.

#### 2.2.1 Single‐Factor Optimization of Extraction Conditions

The effects of four extraction parameters, including ethanol concentration, shaking temperature, incubation time, and extract concentration, on the TPC and FRAP activity of *A. bettzickiana* (green) were investigated through single‐factor optimization. The effect of ethanol concentration was evaluated over a range of 0%–100% (*v/v*) under fixed conditions of 1 h incubating time, 50°C extraction temperature, and 10 mg/mL extract concentration. The effect of incubating time was subsequently examined over a range of 0.5–4 h under fixed conditions of deionized water as the extraction solvent, 30°C shaking temperature, and 10 mg/mL extract concentration. The effect of shaking temperature was investigated over a range of 30°C–90°C under fixed conditions of deionized water as the extraction solvent, 1 h incubating time, and 10 mg/mL extract concentration. Finally, the effect of extract concentration was assessed over a range of 10–50 mg/mL under fixed conditions of deionized water as the extraction solvent, 1 h incubating time, and 50°C shaking temperature.

#### 2.2.2 Response Surface Methodology

Based on the preliminary extraction conditions that optimized TPC and FRAP activity, RSM optimization using a BBD was conducted by setting four independent variables at three levels (−1, 0, and +1): ethanol concentration ($X_{1}$; 50%, 70%, and 90% *v/v*), shaking temperature ($X_{2}$; 30°C, 50°C, and 70°C), incubation time ($X_{3}$; 2, 4, and 6 h), and extract concentration ($X_{4}$; 10, 20, and 30 mg/mL), to investigate their individual and interactive effects on TPC, TFC, and FRAP activity. The resulting experimental data were fitted to a second‐order polynomial equation, from which the coefficient of determination (*R* ^2^), lack‐of‐fit test, regression coefficients, *F*‐values, and *p*‐values were derived for each response. Additionally, plots comparing experimental and predicted response values, along with three‐dimensional (3D) response surface and contour plots illustrating the pairwise interactions between independent variables, were generated to facilitate model interpretation and optimization.

### 2.3 Quantification and Identification of Phenolic Compounds

The phenolic profiles of *A. bettzickiana* (green) and *A. bettzickiana* (variegated) extracts obtained under optimal extraction conditions were analyzed by LC‐ESI‐MS/MS against 26 authentic phenolic standards, following a previously reported protocol (Sirichai et al. 2022; Thangsiri et al. 2024). Prior to analysis, the extracts were evaporated to dryness using a DTC‐22 diaphragm vacuum pump (EYELA CO. LTD., Shanghai, China), yielding recovery yields of 29.2% and 27.5% for *A. bettzickiana* (green) and *A. bettzickiana* (variegated), respectively. The dried extracts were reconstituted in 62.5% (*v/v*) aqueous ethanol to a final concentration of 10 mg/mL. An aliquot of 10 μL of each reconstituted extract was injected onto a 2.1 mm × 100 mm, 2.6 μm Accucore RP‐MS column (Thermo Fisher Scientific, Bremen, Germany) coupled to an LC‐ESI‐MS/MS system (Thermo Fisher Scientific, Bremen, Germany) comprising a Dionex Ultimate 3000 series ultra‐high‐performance liquid chromatography (UHPLC) system, a TSQ Quantis Triple Quadrupole mass spectrometer, and a diode array detector (DAD). The mobile phase consisted of solvent A (acetonitrile) and solvent B (Milli‐Q water with a resistivity of 18.2 MΩ·cm at 25°C containing 0.1% (*v/v*) formic acid), delivered at a constant flow rate of 0.5 mL/min. The gradient elution program was set as follows: 10% solvent A from 0.0 to 0.1 min, ramped to 80% solvent A from 0.1 to 8.0 min, returned to 10% solvent A from 8.0 to 8.1 min, and held at 10% solvent A from 8.1 to 10.0 min. Identification and quantitative analysis were performed using a Chromeleon 7 chromatography data system (Thermo Fisher Scientific, Bremen, Germany).

Twenty‐six authentic phenolic standards were sourced from various commercial suppliers. Isorhamnetin (≥ 99.0% HPLC) was obtained from Extrasynthese (Genay, France). Rosmarinic acid (≥ 98% HPLC) and vanillic acid (≥ 97% HPLC) were purchased from Sigma‐Aldrich (St. Louis, MO, USA). Hesperidin (> 90.0% HPLC), naringenin (> 93.0% HPLC), cinnamic acid (> 98.0% HPLC), quercetin (> 98.0% HPLC), luteolin (> 98.0% HPLC), myricetin (> 97.0% HPLC), *p*‐coumaric acid (> 98.0% GC), (−)‐epigallocatechin gallate (> 98.0% HPLC), apigenin (> 98.0% HPLC), 4‐hydroxybenzoic acid (> 99.0% GC), 3,4‐dihydroxybenzoic acid (≥ 97%), genistein (> 98.0% HPLC), kaempferol (> 97.0% HPLC), caffeic acid (> 98.0% HPLC), sinapic acid (> 99.0% GC), ferulic acid (> 98.0% GC), chlorogenic acid (> 98.0% HPLC), and syringic acid (> 97.0%) were supplied by Tokyo Chemical Industry (Tokyo, Japan). Gallic acid (97.5%–102.5%), galangin (≥ 98.0% HPLC), and rutin (≥ 94% HPLC) were obtained from Wuhan ChemFaces Biochemical Co. Ltd. (Hubei, China). All chromatographic parameters and validation data for the 26 authentic phenolic standards are provided in Tables S2 and S3.

The TPC, TFC, and TAC of *A. bettzickiana* (green) and *A. bettzickiana* (variegated) extracts obtained under optimal extraction conditions were comparatively determined using spectrophotometric methods, following previously reported protocols without modification (Khemthong et al. 2025). All chemicals and reagents were purchased from Sigma‐Aldrich (St. Louis, MO, USA). Absorbance measurements were recorded and analyzed using a SpectraMax Plus 384 microplate reader (Molecular Devices LLC, Sunnyvale, CA, USA), with data processing performed using SoftMax Pro software (version 6.5.1). TPC was determined using Folin–Ciocalteu's phenol reagent, with a calibration curve established using gallic acid (0–200 μg/mL), yielding the linear regression Equation (1) as follows:

(1) y=0.0072x+0.0538

where *x* is the gallic acid concentration (μg/mL) and *y* is the absorbance at 765 nm. The *R* ^2^ of 0.9950 confirmed the linearity of the calibration curve, and TPC was reported as mg gallic acid equivalent (mg GAE)/g dry sample weight (DW).

TFC was determined using aluminum chloride as the chromogenic reagent, with a calibration curve established using quercetin (0–100 μg/mL), yielding the following linear regression Equation (2) as follows:

(2) y=0.0021x+0.0034

where *x* is the quercetin concentration (μg/mL) and *y* is the absorbance at 510 nm. The *R* ^2^ of 0.9934 confirmed the linearity of the calibration curve, and TFC was reported as mg quercetin equivalent (mg QE)/g DW.

TAC was determined using the pH differential method with sodium acetate buffers at pH 1.0 and pH 4.5, with absorbance measured at 520 nm and 700 nm, following a previously reported protocol (Luu et al. 2023). A calibration curve was established using cyanidin‐3‐*O*‐glucoside (2–60 μg/mL), and TAC was reported as mg cyanidin‐3‐*O*‐glucoside equivalent (mg C3GE)/g DW.

### 2.4 Determination of Antioxidant Capacity

The antioxidant capacities of *A. bettzickiana* (green) and *A. bettzickiana* (variegated) extracts obtained under optimal extraction conditions were comparatively evaluated using DPPH radical scavenging, FRAP, and oxygen radical absorbance capacity (ORAC) assays, following previously reported protocols without modification (Whanmek et al. 2026). The DPPH radical scavenging and FRAP assays operate via a single electron transfer (SET) mechanism, whereby antioxidant capacity is determined through chemically induced color changes and measured as end‐point assays. In contrast, the ORAC assay employs a kinetic measurement approach based on the hydrogen atom transfer (HAT) mechanism, as described in detail below. All assays were conducted using a 96‐well microplate format and measured using a Synergy HT microplate reader equipped with Gen 5 data analysis software (BioTek Instruments Inc., Winooski, VT, USA). Standard curves for all three assays were constructed using a series of 6‐hydroxy‐2,5,8‐tetramethylchroman‐2‐carboxylic acid (Trolox) solutions prepared at varying concentrations, and the results were expressed as micromoles of Trolox equivalent (μmol TE)/g DW.

#### 2.4.1 2,2‐Diphenyl‐1‐Picrylhydrazyl (DPPH) Radical Scavenging Assay

For the DPPH assay, 200 μL of 135 μM DPPH solution in 95% (*v/v*) aqueous ethanol was combined with 22 μL of each sample extract and allowed to react in the dark at 25°C for 30 min. Following incubation, the absorbance of the resulting mixture was measured at 520 nm, and the radical scavenging activity was determined according to Equation (3):

(3) $$\text{DPPH radical scavenging activity}=\left(\frac{∆A-I}{S}\right)\times \frac{F}{\mathrm{DW}}\times \frac{\mathrm{fv}}{1000}$$

where *ΔA* represents the difference between the absorbance values of the blank and the sample, *I* denotes the intercept of the Trolox standard curve, *S* denotes the slope of the Trolox standard curve, *F* is the dilution factor applied to the sample, *DW* is the dry weight of the sample (g), and *fv* is the volume of extraction solvent employed (mL).

#### 2.4.2 Ferric Reducing Antioxidant Power (FRAP) Assay

The FRAP reagent was prepared by combining 300 mM acetate buffer (pH 3.6), 10 mM 2,4,6‐tripyridyl‐*s*‐triazine (TPTZ) dissolved in 40 mM hydrochloric acid (HCl), and 20 mM ferric chloride (FeCl₃) solution in a volumetric ratio of 10:1:1, followed by prewarming to 37°C prior to use. For the assay, 150 μL of the freshly prepared FRAP reagent was combined with 20 μL of each sample extract, and the resulting mixture was allowed to react at 25°C for 8 min. The absorbance of the reaction mixture was subsequently recorded at 595 nm, and the FRAP values were determined according to Equation (3) as described above.

#### 2.4.3 Oxygen Radical Absorbance Capacity (ORAC) Assay

For the ORAC assay, 150 μL of 30 mM fluorescein solution was combined with 25 μL of each sample extract and pre‐incubated at 37°C for 30 min. The reaction was subsequently initiated by the addition of 25 μL of 19.1 mM 2,2′‐azobis(2‐amidinopropane) dihydrochloride (AAPH) prepared in 75 mM potassium phosphate buffer (pH 7.4). The decay in fluorescence intensity was then monitored kinetically over a 90 min period at an excitation wavelength of 485 nm and an emission wavelength of 528 nm. The ORAC values were subsequently calculated from the net area under the fluorescence decay curve using Equation (4):

(4) $$\text{Area under the curve}\;\left(\mathrm{AUC}\right)=\left(0.5+f_{1}/f_{0}+f_{2}/f_{0}+f_{3}/f_{0}+\ldots +\left(0.5\right)f_{i}/f_{0}\right)\times \mathrm{CT}$$

where *f₀* denotes the initial fluorescence intensity recorded at 0 min, *fᵢ* denotes the fluorescence intensity measured at the *i*‐th min, and *CT* represents the cycle time (min).

### 2.5 Statistical Analysis

All experiments were performed using three independent sample sets (*n* = 3), with each measurement carried out in triplicate, with the exception of LC‐ESI‐MS/MS analysis, for which each of the three independent sample sets was analyzed once, without technical replication. Results are reported as mean ± standard deviation (SD). Two distinct levels of replication were applied in this study. Biological replicates, referred to throughout the manuscript as independent sample sets (*n* = 3), consisted of three separate batches of freeze‐dried *A. bettzickiana* plant powder prepared independently from plant material collected at the same location and time, but processed through the full sample preparation procedure independently of one another; each biological replicate therefore represented a genuinely independent experimental unit. Technical replicates, referred to throughout the manuscript as triplicate measurements, consisted of three repeated analytical measurements performed on the same extract solution derived from each biological replicate, and were intended to assess analytical precision and reproducibility rather than biological variability. BBD‐based RSM analyses were conducted using Design‐Expert software (version 13.0.5.0, Stat‐Ease Inc., Minneapolis, MN, USA). Statistical comparisons between the experimental results of *A. bettzickiana* (green) and *A. bettzickiana* (variegated) were determined using an unpaired *t*‐test, with statistical significance defined at *p* < 0.05. Prior to *t*‐testing, normality was assessed using the Shapiro–Wilk test and variance homogeneity using Levene's test for each parameter; Welch's correction was applied where variances were unequal (see Table S4 for parameter‐specific results).

## 3 Results

### 3.1 Preliminary Extraction Conditions

The extraction conditions for *A. bettzickiana* (green) were systematically investigated using a single‐factor approach, in which extract concentration, shaking temperature, incubation time, and ethanol concentration were varied individually to evaluate the influence of each independent variable on TPC and FRAP activity (Table 1). The results were subsequently used to establish appropriate variable ranges prior to the application of RSM. As the first variable examined, extract concentration (10–50 mg/mL) was evaluated under controlled conditions comprising an incubation time of 1 h, a shaking temperature of 50°C, and deionized water as the extraction solvent. Maximum TPC and FRAP activity were obtained at an extract concentration of 10 mg/mL, beyond which both responses declined progressively with increasing extract concentration. The effect of shaking temperature (30°C–90°C) was subsequently evaluated under controlled conditions of 1 h incubation time, deionized water as the extraction solvent, and an extract concentration of 10 mg/mL. Maximum TPC was recorded at 30°C, while optimal FRAP activity was observed within the range of 30°C–50°C. A consistent decline in both TPC and FRAP activity was observed as shaking temperature increased beyond 50°C. The effect of incubation time (0.5–4 h) was then investigated under controlled conditions comprising deionized water as the extraction solvent, a shaking temperature of 30°C, and an extract concentration of 10 mg/mL. Both TPC and FRAP activity were maximized within the first 0.5 h of extraction, after which a progressive decline was observed with increasing incubation time. Finally, the effect of ethanol concentration was examined using a fixed incubation time of 1 h, a shaking temperature of 50°C, and an extract concentration of 10 mg/mL. Maximum TPC and FRAP activity were obtained with 80% (*v/v*) aqueous ethanol as the extraction solvent. Ethanol concentrations either below or above this optimum yielded comparatively lower TPC and FRAP activity, suggesting that 80% (*v/v*) aqueous ethanol represents the most effective solvent concentration for the extraction of phenolic compounds from *A. bettzickiana* (green).

**TABLE 1:** Single‐factor effects of varying extraction parameters on the total phenolic content (TPC) and ferric reducing antioxidant power (FRAP) activity of *Alternanthera bettzickiana* (green).

Independent variables		Dependent variables		Controlled variables
		TPC (mg GAE/g DW)	FRAP activity (μmol TE/g DW)	
Extract Concentrations (mg/mL)	10	6.24 ± 0.17^a^	31.23 ± 0.48^a^	Incubating time 1 h Solvent deionized water Shaking temperature 50°C
	20	5.85 ± 0.23^b^	18.07 ± 0.53^b^	
	30	6.00 ± 0.35^b^	18.02 ± 1.24^b^	
	40	5.47 ± 0.21^c^	15.68 ± 0.91^c^	
	50	5.56 ± 0.12^c^	15.15 ± 0.74^c^	
Shaking Temperature (°C)	30	7.06 ± 0.18^a^	21.50 ± 1.02^a^	Incubating time 1 h Solvent deionized water Extract concentration 10 mg/mL
	50	6.91 ± 0.14^b^	21.86 ± 0.93^a^	
	70	4.23 ± 0.07^d^	13.27 ± 0.90^b^	
	90	4.88 ± 0.10^c^	13.33 ± 0.72^b^	
Incubating Time (h)	0.5	7.12 ± 0.18^a^	19.75 ± 0.88^a^	Solvent deionized water Shaking temperature 30°C Extract concentration 10 mg/mL
	1	6.64 ± 0.11^b^	17.88 ± 1.18^b^	
	2	5.89 ± 0.11^c^	14.74 ± 0.76^c^	
	4	5.63 ± 0.30^d^	13.98 ± 0.95^c^	
Ethanol Concentration (% *v/v*)	0	6.47 ± 0.32^b^	8.55 ± 0.36^e^	Incubating time 1 h Shaking temperature 50°C Extract concentration 10 mg/mL
	20	6.35 ± 0.21^c^	12.26 ± 0.36^b^	
	40	6.14 ± 0.14^d^	10.93 ± 0.69^c^	
	60	6.65 ± 0.14^b^	11.24 ± 0.31^c^	
	80	6.84 ± 0.19^a^	22.89 ± 0.80^a^	
	100	2.64 ± 0.17^e^	10.18 ± 0.20^d^	

*Note:* All values are given as mean ± standard deviation (SD) of triplicate measurements conducted on three independent samples (*n* = 3). Statistically significant differences in TPC and FRAP activity were determined at *p* < 0.05 using one‐way analysis of variance (ANOVA) followed by Duncan's multiple range test, with significant differences denoted by different lowercase letters.

Abbreviations: DW, dry weight; GAE, gallic acid equivalent; TE, Trolox equivalent.

### 3.2 Extraction Conditions by Response Surface Methodology (RSM)

The results presented in Table 1 demonstrated that ethanol concentration, shaking temperature, incubation time, and extract concentration each significantly contributed to the yield of phenolics and FRAP activities, indicating that optimal extraction conditions could be determined by simultaneously evaluating these factors using a Box–Behnken design (BBD). Accordingly, the independent variables were set at three levels (−1, 0, and +1), corresponding to ethanol concentration (X_1_; 50%, 70%, and 90% *v/v*), shaking temperature (X_2_; 30°C, 50°C, and 70°C), incubation time (X_3_; 2, 4, and 6 h), and extract concentration (X_4_; 10, 20, and 30 mg/mL), yielding a total of 27 randomized experimental runs, the results of which are presented in Table 2. The experimental TPC values ranged from 3.69 to 6.68 mg GAE/g DW, with the highest value recorded at $X_{1}$ of 90% (*v/v*) aqueous ethanol, $X_{2}$ of 50°C, $X_{3}$ of 4 h, and $X_{4}$ of 10 mg/mL, whereas the corresponding predicted TPC values ranged from 4.11 to 6.22 mg GAE/g DW, with the highest predicted value obtained at $X_{1}$ of 70% (*v/v*) aqueous ethanol, $X_{2}$ of 70°C, $X_{3}$ of 4 h, and $X_{4}$ of 10 mg/mL. Similarly, experimental TFC values ranged from 0 to 27.59 mg QE/g DW, with the maximum observed under the same optimized extraction conditions as TPC, while the predicted TFC values ranged from −0.09 to 24.45 mg QE/g DW, with the highest predicted value obtained under identical conditions. The TFC model generated slightly negative predicted values (−0.09 and −0.10 mg QE/g DW) at runs 8 and 15, where experimental TFC approached zero. These are chemically implausible artifacts of the quadratic model near the experimental boundary, consistent with its overall lack‐of‐fit (*p* < 0.0001). Experimental FRAP activity ranged from 7.79 to 34.09 μmol TE/g DW, reaching its maximum under the same optimized extraction conditions as TPC and TFC, while the corresponding predicted FRAP activity ranged from 7.92 to 34.38 μmol TE/g DW, with the highest value likewise recorded under the same conditions.

**TABLE 2:** Coded and uncoded levels of independent variables, including ethanol concentration ($X_{1}$), shaking temperature ($X_{2}$), incubation time ($X_{3}$), and extract concentration ($X_{4}$), along with the corresponding dependent variables, including total phenolic content (TPC), total flavonoid content (TFC), and ferric reducing antioxidant power (FRAP) activity, of *Alternanthera bettzickiana* (green) derived from the Box–Behnken design (BBD).

Run	$X_{1}$: ethanol concentration (% *v/v*)		$X_{2}$: temperature (°C)		$X_{3}$: time (h)		$X_{4}$: extract concentration (mg/mL)		TPC (mg GAE/g DW)		TFC (mg QE/g DW)		FRAP activity (μmol TE/g DW)	
	Coded	Uncoded	Coded	Uncoded	Coded	Uncoded	Coded	Uncoded	Experimental	Predicted	Experimental	Predicted	Experimental	Predicted
1	0	70	−1	30	0	4	−1	10	5.25 ± 0.12^de^	4.92	0.91 ± 0.11^lm^	3.13	19.95 ± 1.25^d^	19.88
2	0	70	0	50	0	4	0	20	5.07 ± 0.11^ef^	5.24	0.92 ± 0.01^lm^	1.35	15.16 ± 0.47^fg^	15.95
3	1	90	−1	30	0	4	0	20	4.55 ± 0.10^hij^	4.87	16.60 ± 0.86^e^	18.81	27.95 ± 0.69^b^	27.43
4	1	90	1	70	0	4	0	20	5.39 ± 0.16^d^	5.73	19.09 ± 1.30^c^	20.18	27.71 ± 1.71^b^	27.18
5	−1	50	0	50	0	4	1	30	4.14 ± 0.07^k^	4.11	4.29 ± 0.09^i^	7.59	9.11 ± 0.23^lmn^	9.12
6	1	90	0	50	−1	2	0	20	4.96 ± 0.33^fg^	5.81	21.81 ± 1.33^b^	21.30	27.67 ± 1.57^b^	29.07
7	−1	50	1	70	0	4	0	20	4.47 ± 0.07^ij^	5.29	7.00 ± 0.31^h^	6.52	10.00 ± 0.15^kl^	10.07
8	0	70	0	50	−1	2	1	30	4.81 ± 0.36^gh^	4.92	0.00 ± 0.00^m^	−0.10	13.98 ± 1.31^ghi^	13.18
9	0	70	1	70	1	6	0	20	5.11 ± 0.15^ef^	5.61	1.31 ± 0.15^l^	1.54	13.78 ± 0.49^ghi^	14.22
10	0	70	1	70	0	4	1	30	4.57 ± 0.04^hij^	5.02	2.51 ± 0.08^jk^	1.28	12.91 ± 0.42^i^	13.90
11	0	70	1	70	−1	2	0	20	5.16 ± 0.11^def^	5.89	2.46 ± 0.36^jk^	2.32	15.85 ± 0.83^f^	15.99
12	0	70	0	50	0	4	0	20	5.10 ± 0.10^ef^	5.24	0.89 ± 0.13^lm^	1.35	16.04 ± 0.21^f^	15.95
13	−1	50	−1	30	0	4	0	20	4.50 ± 0.17^ij^	4.43	4.50 ± 0.34^i^	5.16	7.79 ± 0.16^n^	7.92
14	0	70	0	50	−1	2	−1	10	5.70 ± 0.07^c^	5.70	1.63 ± 0.11^jkl^	3.80	20.54 ± 0.32^d^	19.98
15	0	70	−1	30	−1	2	0	20	4.43 ± 0.16^ij^	4.83	0.00 ± 0.00^m^	−0.32	13.40 ± 0.47^hi^	13.24
16	0	70	0	50	1	6	−1	10	5.99 ± 0.11^b^	5.59	1.80 ± 0.23^jkl^	3.61	19.77 ± 0.87^d^	20.14
17	0	70	0	50	0	4	0	20	5.17 ± 0.05^def^	5.24	0.88 ± 0.12^lm^	1.35	16.31 ± 1.00^f^	15.95
18	1	90	0	50	0	4	1	30	4.37 ± 0.14^jk^	5.19	11.68 ± 0.29^f^	16.24	24.28 ± 1.32^c^	24.16
19	1	90	0	50	1	6	0	20	4.93 ± 0.05^fg^	4.96	18.06 ± 0.42^d^	17.49	27.57 ± 1.97^b^	27.54
20	0	70	−1	30	1	6	0	20	4.79 ± 0.13^gh^	4.94	1.33 ± 0.05^l^	1.45	14.90 ± 0.37^fgh^	15.06
21	0	70	−1	30	0	4	1	30	3.69 ± 0.06^l^	4.60	2.60 ± 0.14^j^	−0.09	9.81 ± 0.30^lm^	10.34
22	−1	50	0	50	−1	2	0	20	4.56 ± 0.08^hij^	4.60	1.83 ± 0.07^jkl^	3.34	8.47 ± 0.33^mn^	9.21
23	1	90	0	50	0	4	−1	10	6.68 ± 0.21^a^	5.31	27.59 ± 1.64^a^	24.45	34.09 ± 0.55^a^	34.38
24	−1	50	0	50	1	6	0	20	4.67 ± 0.03^hi^	5.28	6.71 ± 0.14^h^	8.14	11.37 ± 0.19^jk^	10.78
25	0	70	0	50	1	6	1	30	4.16 ± 0.08^k^	4.86	1.54 ± 0.17^kl^	1.08	12.94 ± 0.69^i^	13.06
26	−1	50	0	50	0	4	−1	10	5.13 ± 0.02^ef^	5.51	10.23 ± 0.41^g^	5.80	12.41 ± 0.46^ij^	12.78
27	0	70	1	70	0	4	−1	10	6.11 ± 0.18^b^	6.22	1.02 ± 0.1 1^l^	4.48	17.88 ± 0.29^e^	18.24

*Note:* All values are given as mean ± standard deviation (SD) of triplicate measurements conducted on three independent samples (*n* = 3). Statistically significant differences in the same measurement among experimental runs were determined at *p* < 0.05 using one‐way analysis of variance (ANOVA) followed by Duncan's multiple range test, with significant differences denoted by different lowercase letters. The predicted values were calculated using the following equations:
$$\mathrm{TPC}:Y=0.3986+0.0735\;X_{1}+0.0532\;X_{2}+0.6015\;X_{3}-0.0772\;X_{4}-0.0000\;X_{1}X_{2}-0.0096\;X_{1}X_{3}+0.0016\;X_{1}X_{4}-0.0024\;X_{2}X_{3}-0.0011\;X_{2}X_{4}+0.0006\;X_{3}X_{4}-0.0004\;X_{1}^{2}-0.0000\;X_{2}^{2}+0.0197\;X_{3}^{2}-0.0005\;X_{4}^{2}$$


$$\mathrm{TFC}:Y=85.7675-3.1555\;X_{1}+0.0977\;X_{2}+4.5469\;X_{3}+0.3049\;X_{4}-0.0000\;X_{1}X_{2}-0.0538\;X_{1}X_{3}-0.0125\;X_{1}X_{4}-0.0160\;X_{2}X_{3}+0.00002\;X_{2}X_{4}+0\;.0171\;X_{3}X_{4}+0.0283\;X_{1}^{2}+0.0000\;X_{2}^{2}-0.0249\;X_{3}^{2}+0.0085\;X_{4}^{2}$$


$$\text{FRAP activity}:Y=5.6784-0.4016\;X_{1}+0.3785\;X_{2}+2.8731\;X_{3}-0.4044\;X_{4}-0.0015\;X_{1}X_{2}-0.0194\;X_{1}X_{3}-0.0082\;X_{1}X_{4}-0.0224\;X_{2}X_{3}+0.0065\;X_{2}X_{4}-0.0034\;X_{3}X_{4}+0.0084\;X_{1}^{2}-0.0029\;X_{2}^{2}-0.0401\;X_{3}^{2}+0.0080\;X_{4}^{2}$$
where $X_{1}$, $X_{2}$, $X_{3}$, and $X_{4}$ represent the independent variables ethanol concentration (% *v/v*), shaking temperature (°C), incubation time (h), and extract concentration (mg/mL), respectively. For completeness and reproducibility, the full second‐order polynomial equations are reported for all three responses; the statistical significance of each individual linear, interaction, and quadratic term is presented in Table 3 and should be consulted when interpreting the relative contribution of each term. Negative predicted TFC values (−0.09 to −0.10 mg QE/g DW) at runs 8 and 15 are a mathematical artifact of the quadratic model at the lower boundary of the experimental domain and do not reflect physically meaningful negative flavonoid content.

Abbreviations: DW, dry weight; GAE, gallic acid equivalent; QE, quercetin equivalent; TE, Trolox equivalent.

**FIGURE 2:**
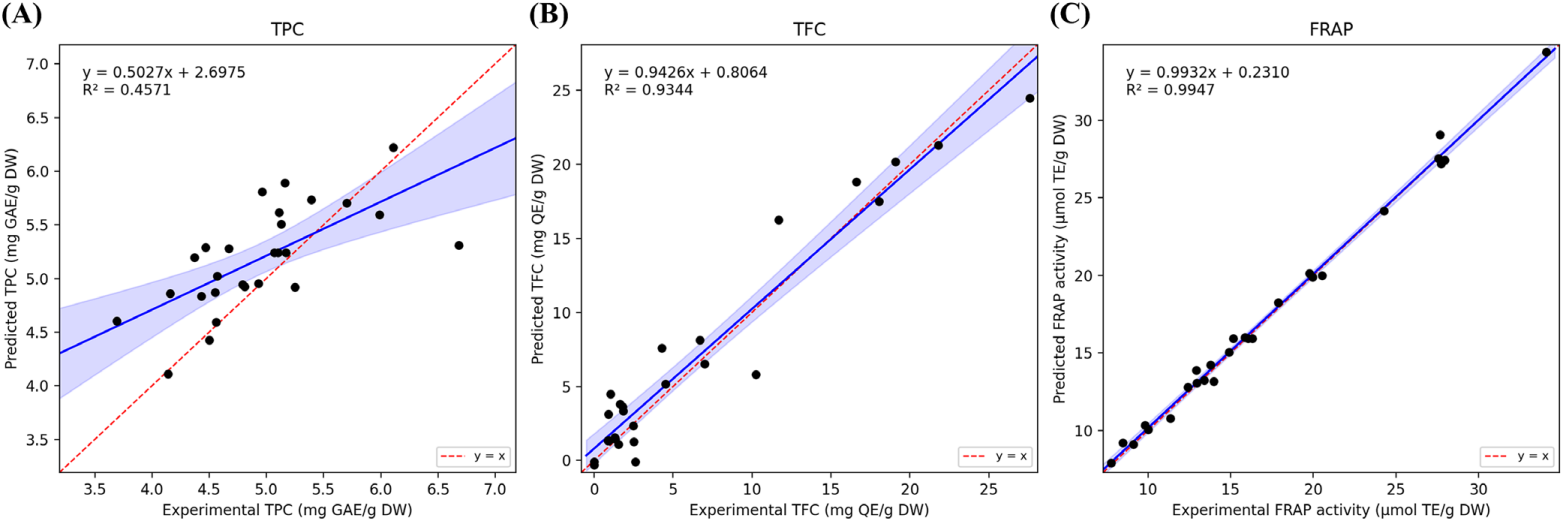
Correlation between experimental and predicted values, with fitted regression equations and 95% confidence intervals, for (A) total phenolic content (TPC), (B) total flavonoid content (TFC), and (C) ferric reducing antioxidant power (FRAP) activity of *Alternanthera bettzickiana* (green) under the extraction conditions tested.

The ANOVA results of the BBD‐derived second‐order polynomial models for the extraction of *A. bettzickiana* (green) with respect to TPC, TFC, and FRAP activity are presented in Table 3. For TPC, the regression model was not statistically significant (*F*‐value = 1.51 and *p* = 0.2409), with an *R* ^2^ of 0.6375 and an adjusted *R* ^2^ of 0.2147, indicating that the model accounted for approximately 63.8% of the total variation while explaining only 21.5% of the variability in the TPC response after adjustment for the number of predictors, thereby reflecting a poor predictive fit. Furthermore, the lack‐of‐fit was statistically significant (*p* = 0.0215), indicating systematic deviations between the model predictions and the observed data, as illustrated in Figure 2A. Consequently, the quadratic model developed for TPC was deemed inadequate for optimization purposes.

**TABLE 3:** Analysis of variance (ANOVA) of the Box–Behnken design (BBD) for the effects of independent variables on total phenolic content (TPC), total flavonoid content (TFC), and ferric reducing antioxidant power (FRAP) activity of *Alternanthera bettzickiana* (green) extraction.

Source	TPC						TFC						FRAP activity					
	Sum of squares	df	Mean square	*F*‐value	*p*	Sig	Sum of squares	df	Mean square	*F*‐value	*p*	Sig	Sum of squares	df	Mean square	*F*‐value	*p*	Sig
Model	5.10	14	0.3643	1.51	0.2409	ns	1456.37	14	104.03	12.31	< 0.0001	****	1275.09	14	91.08	165.53	< 0.0001	****
X_1_	0.7450	1	0.7450	3.08	0.1046		537.07	1	537.07	63.53	< 0.0001	****	1008.88	1	1008.88	1833.65	< 0.0001	****
X_2_	1.10	1	1.10	4.57	0.0538		4.27	1	4.27	0.5054	0.4907		1.56	1	1.56	2.83	0.1185	
X_3_	0.0133	1	0.0133	0.0552	0.8182		0.7301	1	0.7301	0.0864	0.7739		0.0021	1	0.0021	0.0039	0.9514	
X_4_	1.77	1	1.77	7.33	0.0191	*	35.23	1	35.23	4.17	0.0638		144.14	1	144.14	261.98	< 0.0001	****
X_1_X_2_	0.0000	1	0.0000	0.0001	0.9921		0.0001	1	0.0001	0.0000	0.9973		1.50	1	1.50	2.73	0.1245	
X_1_X_3_	0.5852	1	0.5852	2.42	0.1456		18.53	1	18.53	2.19	0.1645		2.40	1	2.40	4.37	0.0586	
X_1_X_4_	0.4032	1	0.4032	1.67	0.2207		24.85	1	24.85	2.94	0.1121		10.63	1	10.63	19.32	0.0009	***
X_2_X_3_	0.0361	1	0.0361	0.1494	0.7059		1.64	1	1.64	0.1938	0.6676		3.22	1	3.22	5.86	0.0323	*
X_2_X_4_	0.2070	1	0.2070	0.8568	0.3729		0.0100	1	0.0100	0.0012	0.9731		6.66	1	6.66	12.10	0.0046	**
X_3_X_4_	0.0006	1	0.0006	0.0026	0.9603		0.4692	1	0.4692	0.0555	0.8177		0.0182	1	0.0182	0.0331	0.8586	
X_1_^2^	0.1281	1	0.1281	0.5303	0.4805		681.06	1	681.06	80.57	< 0.0001	****	60.33	1	60.33	109.65	< 0.0001	****
X_2_^2^	0.0024	1	0.0024	0.0100	0.9221		0.0003	1	0.0003	0.0000	0.9951		7.31	1	7.31	13.28	0.0034	**
X_3_^2^	0.0331	1	0.0331	0.1369	0.7178		0.0529	1	0.0529	0.0063	0.9383		0.1372	1	0.1372	0.2494	0.6265	
X_4_^2^	0.0108	1	0.0108	0.0447	0.8361		3.86	1	3.86	0.4563	0.5122		3.40	1	3.40	6.18	0.0287	*
Residual	2.90	12	0.2416				101.44	12	8.45				6.60	12	0.5502			
Lack‐of‐Fit	2.89	10	0.2887	45.82	0.0215	*	101.44	10	10.14	23409.50	< 0.0001	****	5.88	10	0.5875	1.62	0.4421	ns
Pure Error	0.0126	2	0.0063				0.0009	2	0.0004				0.7274	2	0.3637			
Cor Total	8.00	26					1557.81	26					1281.69	26				
*R* ^2^					0.6375						0.9349						0.9948	
*R* ^2^ adjusted					0.2147						0.8589						0.9888	

*Note:* Statistical analyses were analyzed using one‐way ANOVA with * denotes *p* < 0.05, ** denotes *p* < 0.01, *** denotes *p* < 0.001, and **** denotes *p* < 0.0001.

Abbreviations: $X_{1}$, ethanol concentration; $X_{2}$, shaking temperature; $X_{3}$, incubating time; $X_{4}$, extract concentration; ns, not significance.

In contrast, the fitted model for TFC was highly significant (*F*‐value = 12.31 and *p* < 0.0001), demonstrating a strong overall relationship between the independent variables and the TFC response. The model accounted for approximately 93.5% of the total variation, as reflected by an *R* ^2^ of 0.9349 and an adjusted *R* ^2^ of 0.8589, indicating a satisfactory model fit with minimal deviation between the experimental and predicted values, as shown in Figure 2B. Nevertheless, the lack‐of‐fit remained highly significant (*p* < 0.0001), suggesting that systematic deviations not fully captured by the quadratic model structure were present. This was reflected in several run‐level discrepancies between experimental and predicted values: at Run 21, the experimental TFC value (2.60 mg QE/g DW) diverged from the predicted value (−0.09 mg QE/g DW), the latter reflecting the known tendency of second‐order polynomial models to generate physically implausible negative predictions near the domain boundaries, while a similar deviation occurred at Run 23 (27.59 vs. 24.45 mg QE/g DW). These discrepancies likely reflect non‐linear parameter interactions not fully captured by the quadratic model, matrix interference in the aluminum chloride assay, and inherent biological variability among samples. Given these limitations, the TFC model was not used for optimization.

The fitted model for FRAP activity demonstrated an excellent fit (*F*‐value = 165.53 and *p* < 0.0001), with an *R* ^2^ of 0.9948 and an adjusted *R* ^2^ of 0.9888, indicating that approximately 99.5% of the total variation in FRAP activity was explained by the model (Figure 2C). Notably, the lack‐of‐fit was non‐significant (*p* = 0.4421), confirming that the model adequately represented the true relationship between the independent variables and the response. This model is therefore considered the most reliable among the three responses and is well‐suited for optimization purposes. With respect to the individual model terms, the *p*‐values of the two linear coefficients for ethanol concentration and extract concentration were less than 0.001, indicating a dominant linear influence of these two factors on FRAP activity. In contrast, the linear coefficients for shaking temperature and incubation time were non‐significant (*p* = 0.1185 and *p* = 0.9514, respectively), suggesting limited overall influence on the measured response within the experimental range tested. Among the pairwise interaction terms, the interactions between ethanol concentration and extract concentration ($X_{1}X_{4}$), shaking temperature and incubation time ($X_{2}X_{3}$), and shaking temperature and extract concentration ($X_{2}X_{4}$) significantly affected FRAP activity, with the ethanol concentration–extract concentration interaction yielding the lowest *p*‐value (*p* = 0.0009), indicating its particularly prominent role in modulating antioxidant capacity. All remaining interaction terms were non‐significant, indicating the absence of meaningful two‐factor interactions for those variable combinations. Among the quadratic terms, those corresponding to ethanol concentration ($X_{1}^{2}$), shaking temperature ($X_{2}^{2}$), and extract concentration ($X_{4}^{2}$) were statistically significant (*p* < 0.0001, *p* = 0.0034, and *p* = 0.0287, respectively), suggesting nonlinear behavior of these factors in the antioxidant response. Overall, a multiple regression analysis described the relationship between these independent variables and the antioxidant variables as a second‐order polynomial equation as follows.

$$\begin{array}{cc} Y= & 5.6784-0.4016\;X_{1}+0.3785\;X_{2}+2.8731\;X_{3}-0.4044\;X_{4}\\ & -0.0015\;X_{1}X_{2}-0.0194\;X_{1}X_{3}-0.0082\;X_{1}X_{4}-0.0224\;X_{2}X_{3}\\ & +0.0065\;X_{2}X_{4}-0.0034\;X_{3}X_{4}+0.0084\;X_{1}^{2}-0.0029\;X_{2}^{2}\\ & -0.0401\;X_{3}^{2}+0.0080\;X_{4}^{2} \end{array}$$

where Y is the predicted FRAP activity (μmol TE/g DW), $X_{1}$ is the ethanol concentration (% *v/v*), $X_{2}$ is the shaking temperature (°C), $X_{3}$ is the incubating time (h), and $X_{4}$ is the extract concentration (mg/mL).

**FIGURE 3:**
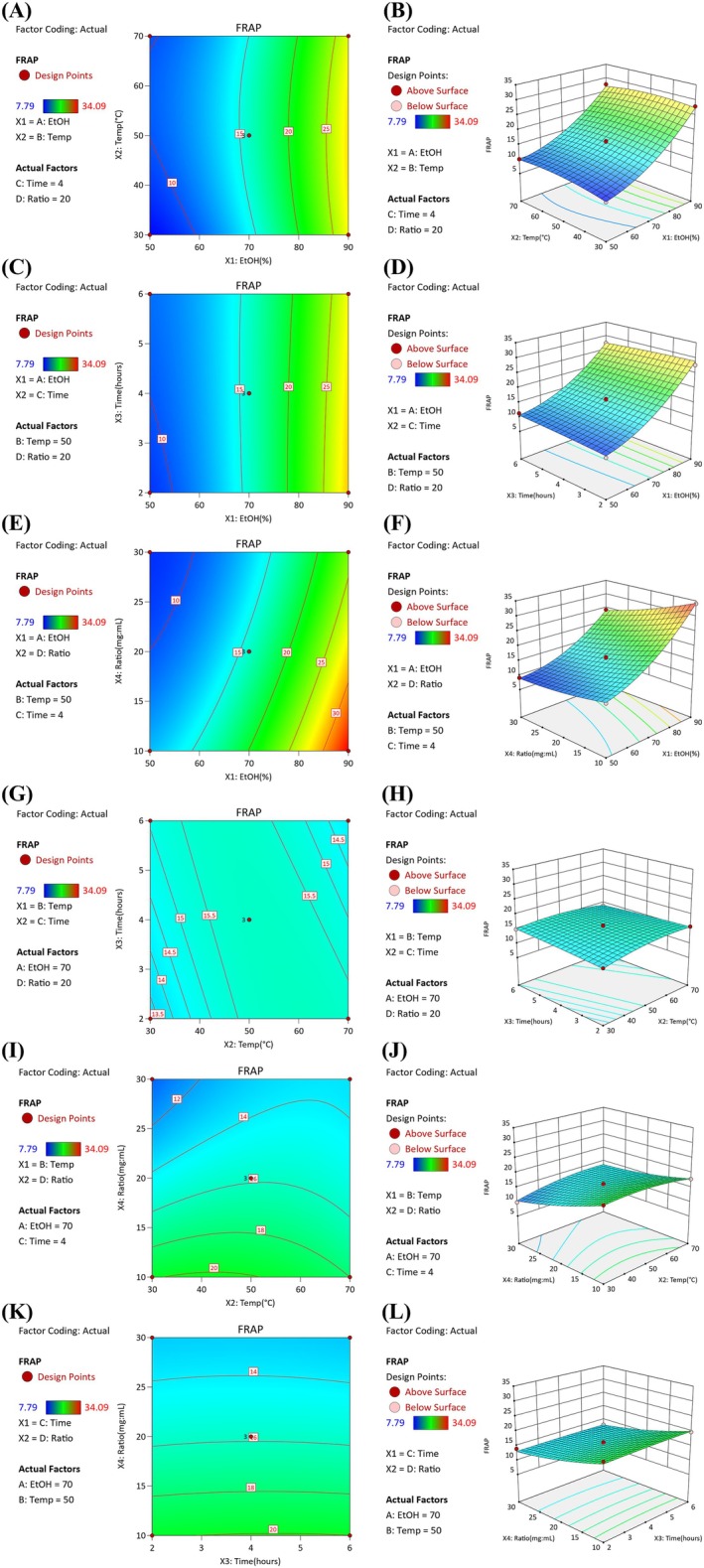
Optimization of *Alternanthera bettzickiana* (green) extract using response surface methodology (RSM), represented as contour plots (A, C, E, G, I, K) and three‐dimensional response surface plots (B, D, F, H, J, L) illustrating the effects of ethanol concentration (*X_1_*), shaking temperature (*X_2_*), incubating time (*X_3_*), and extract concentration (*X_4_*) on ferric reducing antioxidant power (FRAP) activity. Red, green, and blue regions indicate high, intermediate, and low FRAP activities, respectively.

To further investigate the strengths of the pairwise interactions between independent variables including ethanol concentration ($X_{1}$), shaking temperature ($X_{2}$), incubating time ($X_{3}$), and extract concentration ($X_{4}$) with respect to FRAP activity, contour and three‐dimensional (3D) response surface plots were generated for all six pairwise combinations and are presented in Figure 3A–L. The pairwise interaction between ethanol concentration and shaking temperature ($X_{1}X_{2}$) indicated that increasing ethanol concentration progressively elevated FRAP activity, while increasing shaking temperature enhanced FRAP activity only up to approximately 50°C, beyond which a decline was observed (Figure 3A,B). Notably, ethanol concentration exerted a substantially greater influence on FRAP activity than shaking temperature. A similar trend was observed for the pairwise interaction between ethanol concentration and incubation time ($X_{1}X_{3}$), wherein incubation time exhibited a lesser effect on FRAP activity than ethanol concentration, and shorter incubation times yielded only marginally higher FRAP activity compared to longer durations (Figure 3C,D). A more pronounced effect was observed in the pairwise interaction between ethanol concentration and extract concentration ($X_{1}X_{4}$), where lower extract concentrations in combination with higher ethanol concentrations clearly yielded greater FRAP activity (Figure 3E,F). In contrast, the pairwise interactions between shaking temperature and incubation time ($X_{2}X_{3}$, Figure 3G,H), shaking temperature and extract concentration ($X_{2}X_{4}$, Figure 3I,J), and incubation time and extract concentration ($X_{3}X_{4}$, Figure 3K,L) exerted comparatively little influence on FRAP activity, although elevated FRAP activity was noted at low extract concentrations combined with an intermediate shaking temperature. Collectively, the response surface data confirmed the predominant influence of ethanol concentration and extract concentration on FRAP activity, while shaking temperature and incubation time contributed minimally within the experimental range tested. Statistically, these visual trends were largely corroborated by the ANOVA results (Table 3), although two interactions reached statistical significance despite their modest practical effect sizes: the ethanol concentration–extract concentration interaction ($X_{1}X_{4}$) was the most significant term (*p* = 0.0009), consistent with the pronounced antioxidant response in Figure 3E,F, reflecting enhanced phenolic solubilization at high ethanol concentration combined with greater solid‐to‐liquid interfacial area at low extract concentration. The shaking temperature–incubation time ($X_{2}X_{3}$, *p* = 0.0323) and shaking temperature–extract concentration ($X_{2}X_{4}$, *p* = 0.0046) interactions also reached statistical significance despite their comparatively modest visual influence on FRAP activity noted above, consistent with the trade‐off between improved mass transfer at intermediate temperatures and thermal degradation of phenolics at higher temperatures. In contrast, the ethanol concentration–shaking temperature ($X_{1}X_{2}$), ethanol concentration–incubation time ($X_{1}X_{3}$), and incubation time–extract concentration ($X_{3}X_{4}$) interactions were non‐significant (*p* > 0.05), consistent with the comparatively flat response surfaces observed in their respective plots. The optimized extraction conditions for maximum FRAP activity were determined to be 89.8% (*v/v*) aqueous ethanol, 47.6°C extraction temperature, 2 h incubation time, and 10.5 mg/mL extract concentration. For practical laboratory application, these conditions were adjusted to 90% (*v/v*) aqueous ethanol, 50°C extraction temperature, 2 h incubation time, and 10 mg/mL extract concentration. Under these optimized conditions, the predicted FRAP activity was 34.91 μmol TE/g DW, which closely corresponded to the experimentally determined value of 37.38 μmol TE/g DW, corresponding to a prediction error of approximately 7.08%, thereby validating the reliability of the model. The 95% prediction interval for the optimized conditions, as generated by the Design‐Expert software, was 32.36 to 37.23 μmol TE/g DW. The experimental validation value fell marginally above the upper bound of this interval, indicating that the model slightly underestimated FRAP activity at the optimum; this minor deviation is within the expected range of experimental variability and does not compromise the overall adequacy of the validated model.

### 3.3 Phenolic Profile and Contents

**FIGURE 4:**
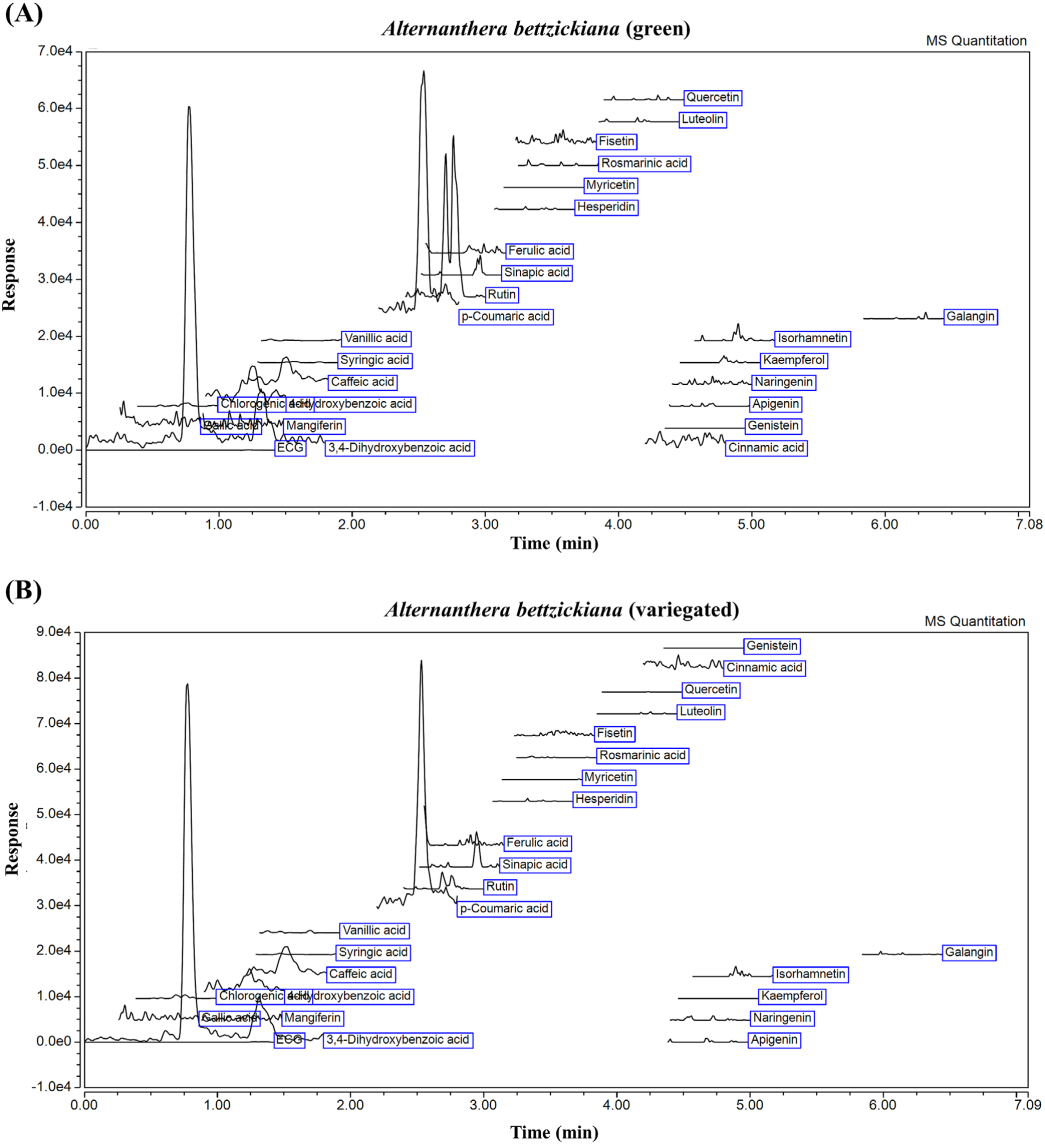
Liquid chromatography coupled with electrospray ionization‐tandem mass spectrometry (LC‐ESI‐MS/MS) chromatograms of aqueous ethanolic extracts of (A) *A. bettzickiana* (green) and (B) *A. bettzickiana* (variegated) obtained under optimized extraction conditions.

Under optimized extraction conditions, the phenolic profiles of aqueous ethanolic extracts of *A. bettzickiana* (green) and *A. bettzickiana* (variegated) were characterized by LC‐ESI‐MS/MS against 26 authentic phenolic standards, with the results presented in Table 4. Full integration results and chromatograms are provided in Tables S5 and S6, and Figure 4, respectively. Among the identified compounds, *p*‐coumaric acid was the predominant phenolic, detected at concentrations ranging from 187.99 to 196.84 μg/g extract, representing a 1.4‐fold higher content compared to 3,4‐dihydroxybenzoic acid (130.06–137.44 μg/g extract). Overall, slightly higher *p*‐coumaric acid and 3,4‐dihydroxybenzoic acid contents were observed in *A. bettzickiana* (variegated); however, rutin was exclusively detected in *A. bettzickiana* (green) at a trace concentration of 1.98 μg/g extract.

**TABLE 4:** Phenolic profile identified by liquid chromatography coupled with electrospray ionization tandem mass spectrometry (LC‐ESI‐MS/MS) in selective reaction monitoring (SRM) mode, along with total phenolic content (TPC), total flavonoid content (TFC), and total anthocyanin content (TAC) of aqueous ethanolic extracts of *Alternanthera bettzickiana* (green) and *Alternanthera bettzickiana* (variegated) obtained under optimized extraction conditions.

Phenolic identification and quantification	Ion mass	Parent ions (*m*/*z*)	SRM transitions (*m*/*z*) and collision energy (*V*)	RF lens *(V)*	*A. bettzickiana* (green)	*A. bettzickiana* (variegated)
Targeted phenolics by LC‐ESI‐MS/MS (μg/g extract)						
3,4‐Dihydroxybenzoic acid	[M‐H]	152.95	109.113 (14.35 V), 81.042 (20.50 V), 91.042 (24.59 V)	128	130.06 ± 1.31^ns^	137.44 ± 4.81
*p*‐Coumaric acid	[M + H]	165.05	147.054 (11.70 V), 119.113 (19.36 V), 91.125 (25.89 V)	90	187.99 ± 3.16^b^	196.84 ± 2.26^a^
Rutin	[M + H]	611.2	303.13 (20.80), 465.20 (12.71 V)	198	1.98 ± 0.08	ND
TPC (mg GAE/g DW)					4.95 ± 0.39^a^	4.02 ± 0.14^b^
TFC (mg QE/g DW)					25.78 ± 2.30^b^	31.95 ± 1.40^a^
TAC (mg C3GE/g DW)					ND	7.04 ± 0.63

*Note:* All values are given as mean ± standard deviation (SD) of triplicate measurements conducted on three independent samples (*n* = 3). Statistically significant differences in phenolic contents between species were determined at *p* < 0.05 using an unpaired *t*‐test, with significant differences denoted by different lowercase letters. For TPC, TFC and TAC, the mean of the three technical replicates was calculated first for each biological replicate; the reported mean ± SD, as well as all statistical comparisons described below, were then based on the resulting three biological replicate means (*n* = 3) rather than on the nine individual technical measurements, consistent with standard practice for avoiding pseudoreplication. Normality (Shapiro–Wilk test) and variance homogeneity (Levene's test) were confirmed for TPC and TFC, supporting the use of Student's *t*‐test without correction for both parameters. TAC was not statistically compared between species: *A. bettzickiana* (green) yielded negative computed values, reflecting negligible/non‐detectable anthocyanin content (ND) rather than genuine measurements, so normality and variance‐homogeneity testing and *t*‐testing were not applicable for this parameter; only TAC of *A. bettzickiana* (variegated) was reported. For the LC‐ESI‐MS/MS‐quantified compounds (3,4‐dihydroxybenzoic acid and *p*‐coumaric acid), each of the three independent samples was injected once, without technical replication (*n* = 3 measurements per species), in contrast to TPC, TFC, and TAC, which were each measured in triplicate for every independent sample and analyzed as three biological replicate means (*n* = 3; see Section 2.5). Normality and variance homogeneity were nonetheless assessed for the LC‐ESI‐MS/MS compounds (Shapiro–Wilk and Levene's tests, both *p* > 0.05 for each compound), supporting the use of Student's *t*‐test without correction; as with all comparisons in this study, the Shapiro–Wilk test has limited power at *n* = 3 to detect departures from normality, and these results should be interpreted with appropriate caution. Rutin was not detected (ND) in *A. bettzickiana* (variegated) and could therefore not be statistically compared between species.

Abbreviations: C3GE, cyanidin‐3‐*O*‐glucoside equivalent; DW, dry weight; GAE, gallic acid equivalent; ND, not detected; ns, not significant; QE, quercetin equivalent.

Under spectrophotometric measurement, the TPC of the aqueous ethanolic extract of *A. bettzickiana* (green) was marginally higher than that of *A. bettzickiana* (variegated) (4.95 vs. 4.02 mg GAE/g DW), whereas a more distinctly higher TFC was recorded in the latter (31.95 vs. 25.78 mg QE/g DW). Notably, TAC was exclusively detected in *A. bettzickiana* (variegated) (7.04 mg C3GE/g DW), which is consistent with the characteristic red pigmentation observed in its leaves.

### 3.4 Antioxidant Activities

The antioxidant activities of aqueous ethanolic extracts obtained from both *A. bettzickiana* species under optimized extraction conditions were evaluated using three complementary assays: DPPH radical scavenging, FRAP, and ORAC, with the results presented in Table 5. No statistically significant difference in DPPH radical scavenging activity was observed between the two species. In contrast, *A. bettzickiana* (green) exhibited significantly higher FRAP activity, recording a 1.2‐fold increase compared to *A. bettzickiana* (variegated). A more pronounced interspecies difference was observed in ORAC activity, where *A. bettzickiana* (green) demonstrated a 1.4‐fold higher antioxidant capacity relative to *A. bettzickiana* (variegated).

**TABLE 5:** Antioxidant activities of aqueous ethanolic *Alternanthera bettzickiana* extracts obtained under optimized extraction conditions, as evaluated by 2,2‐diphenyl‐1‐picrylhydrazyl (DPPH) radical scavenging, ferric reducing antioxidant power (FRAP), and oxygen radical absorbance capacity (ORAC) assays.

Antioxidant activity (μmol TE/g DW)	*A. bettzickiana* (green)	*A. bettzickiana* (variegated)
DPPH radical scavenging activity	13.75 ± 0.75^ns^	13.40 ± 0.92
FRAP activity	37.38 ± 1.34^a^	32.41 ± 2.42^b^
ORAC activity	235.33 ± 19.31^a^	173.62 ± 16.21^b^

*Note:* All values are given as mean ± standard deviation (SD) of triplicate measurements conducted on three independent samples (*n* = 3). Statistically significant differences in antioxidant activity between species were determined at *p* < 0.05 using an unpaired *t*‐test, with significant differences denoted by different lowercase letters. Normality (Shapiro–Wilk test) and variance homogeneity (Levene's test) were confirmed for DPPH radical scavenging, FRAP, and ORAC activities (all *p* > 0.05), supporting the use of Student's *t*‐test without correction. For all antioxidant activities, the mean of the three technical replicates was calculated first for each biological replicate; the reported mean ± SD and the statistical comparisons above were based on the resulting three biological replicate means (*n* = 3) rather than on the nine individual technical measurements, consistent with standard practice for avoiding pseudoreplication.

Abbreviations: DW, dry weight; ns, not significant; TE, Trolox equivalent.

## 4 Discussion

The ANOVA results for the TPC, TFC, and FRAP models indicate that the reliability of the BBD‐derived response surface model was assay‐dependent. The poor fit of the TPC model, evidenced by its non‐significant regression and significant lack‐of‐fit, suggests that the relationship between the four extraction parameters and Folin–Ciocalteu‐based phenolic quantification is not adequately captured by a second‐order polynomial within the tested experimental range, potentially reflecting matrix‐associated interference from non‐phenolic reducing substances or genuine non‐quadratic behavior of the underlying extraction process. Although the TFC model achieved a statistically significant and high‐*R* ^2^ fit, its persistently significant lack‐of‐fit indicates that some systematic variation remained unexplained, warranting cautious interpretation of TFC‐based predictions. In contrast, the FRAP model exhibited an excellent fit with a non‐significant lack‐of‐fit, indicating that it robustly captured the true relationship between extraction parameters and antioxidant capacity and could therefore be used with confidence to identify the optimized extraction conditions; this was further confirmed by close agreement between the predicted (34.91 μmol TE/g DW) and experimental (37.38 μmol TE/g DW) FRAP activity at the optimized conditions, demonstrating that BBD‐based RSM can reliably model antioxidant extraction from a leafy, phenolic‐rich plant matrix when an appropriate, interference‐resistant response variable is selected. Collectively, these findings offer a broader methodological lesson for future extraction‐optimization studies: colorimetric assays such as the Folin–Ciocalteu (TPC) and aluminum chloride (TFC) methods, while convenient, may be more susceptible to matrix‐specific interferences that compromise model adequacy, whereas electron‐transfer‐based antioxidant assays such as FRAP may serve as more robust surrogate responses for RSM optimization. For future RSM‐based extraction optimization of *A. bettzickiana* or phytochemically similar matrices, researchers encountering non‐significant TPC/TFC models should therefore consider expanding the experimental design (e.g., additional center points or higher‐order terms) or selecting alternative, more model‐compatible response variables such as FRAP. More broadly, the validated extraction protocol and modeling framework established here (ethanol concentration, shaking temperature, incubation time, and extract concentration) could serve as a practical template for optimizing bioactive compound recovery from other underexplored leafy vegetables or phenolic‐rich plant matrices, particularly where no standardized extraction protocol currently exists.

Among the parameters evaluated, ethanol concentration exerted the greatest influence on antioxidant extraction from *A. bettzickiana* . Aqueous ethanol has been widely reported as a more effective solvent for phenolic antioxidant extraction than either absolute ethanol or pure water (Sun et al. 2015; Waszkowiak and Gliszczyńska‐Świgło 2016), an effect attributable to its intermediate polarity. Based on established polarity index values for aqueous ethanol mixtures (Kumoro et al. 2009), 90% (*v/v*) aqueous ethanol yields an estimated polarity index of approximately 5.6, rendering it suitable for the solubilization of phenolic antioxidants with comparable polarity characteristics. Furthermore, ethanol has been reported to inactivate polyphenol oxidase, an enzyme responsible for phenolic degradation upon cell wall disruption during extraction, thereby enhancing the stability of extracted phenolic antioxidants relative to pure aqueous systems (Zhang 2023). The present study identified 90% (*v/v*) aqueous ethanol as optimal for maximizing phenolic antioxidant recovery from *A. bettzickiana* (green), yielding the highest FRAP activity under optimized conditions, consistent with previous reports on antioxidant extraction from related Amaranthaceae members. A study on *A. sessilis* , a closely related species, reported an optimized extraction using ethanol, in which the ethanolic extract exhibited 5.0‐fold higher FRAP activity than water extraction, 3.6‐fold higher than hexane, and 2.5‐fold higher than ethyl acetate extraction (Hazli et al. 2019), further supporting the effectiveness of ethanolic extracted systems in this genus. This is consistent with the intermediate polarity of aqueous ethanol relative to less polar solvents such as hexane and ethyl acetate, which enhances the solubilization of phenolic antioxidants and thereby supports the selection of aqueous ethanol as the optimal extraction solvent in the present study.

Extract concentration was identified as the second most influential parameter affecting antioxidant recovery from *A. bettzickiana* , with a sample concentration of 10 mg/mL determined to be optimal relative to higher concentrations. This is consistent with the mass transfer principle, whereby a lower solid‐to‐liquid ratio increases the interfacial contact area between the solid matrix and solvent, thereby enhancing the driving force for mass transfer of phenolic antioxidants across plant cell walls into the extraction medium (Both et al. 2015). At elevated extract concentrations, solvent saturation and inter‐particle interference may compromise mass transfer efficiency, limiting solvent penetration into the plant matrix and promoting co‐extraction of interfering macromolecules (proteins, polysaccharides) that may compete with or bind phenolic compounds, collectively resulting in reduced extraction yield and diminished antioxidant activity. This pattern is well supported in the literature: a reduction in phenolic yield from grape pomace beyond an optimal solid‐to‐liquid ratio has been attributed to solvent saturation (Pinelo et al. 2005); an inverse relationship between extract concentration and TPC has similarly been reported in RSM‐optimized wheat extracts (Liyana‐Pathirana and Shahidi 2005); and a concentration of 10 mg/mL was likewise identified as optimal for *Aeginetia indica* L. using the extraction protocol adopted in the present study (On‐Nom et al. 2024). Regarding the 10–30 mg/mL range investigated, we acknowledge that the lower boundary was constrained by the preliminary single‐factor experiment, which did not test below 10 mg/mL. Lower concentrations might yield marginally higher recovery, but would require proportionally more raw plant material, which was impractical given the limited availability of *A. bettzickiana* (variegated).

Shaking temperature, identified as a moderately influential parameter, was optimized at 50°C, consistent with the well established positive relationship between temperature and extraction efficiency. Elevated temperatures enhance solute solubility and mass transfer rates through increased diffusion coefficients, reduced solvent viscosity, and disruption of non‐covalent interactions between phenolics and cell wall constituents (cellulose, hemicellulose, pectin), releasing otherwise bound phenolics (Albarri et al. 2023). These effects are well documented: a significant positive correlation between temperature and phenolic yield from brewers spent grain has been reported up to an optimal threshold (Rahman, Malunga, et al. 2021), and increasing extraction temperature from 25°C to 45°C significantly enhanced polyphenol recovery from grape marc due to increased diffusivity and reduced viscosity (Spigno et al. 2007). Nevertheless, excessively high temperatures may accelerate the degradation of thermally labile compounds through oxidation, hydrolysis, and isomerization, with anthocyanins being particularly susceptible to glycosidic bond hydrolysis and flavylium cation ring opening (Antony and Farid 2022). Consistently, the present study observed a progressive decline in TPC and FRAP activity above 50°C, with maximum values at 30 to 50°C, in agreement with reports that phenolic extraction from wheat and black currants similarly peaked at intermediate temperatures before declining due to thermal degradation (Cacace and Mazza 2003; Liyana‐Pathirana and Shahidi 2005). The selected temperature of 50°C therefore represents an optimal balance between maximizing extraction efficiency and preserving the integrity of phenolic antioxidant constituents in *A. bettzickiana*.

Incubation time was found to exert the least influence on antioxidant yield, consistent with the solid–liquid extraction principle whereby solvent diffusion into the plant matrix and subsequent solubilization of target compounds reach equilibrium within a finite period (Niawanti et al. 2022). Beyond this equilibrium point, prolonged extraction does not substantially increase antioxidant yield and may instead promote compound degradation or co‐extraction of undesirable matrix components. An extraction duration of 2 h was therefore determined to be sufficient to achieve near‐complete solubilization of target compounds while minimizing these adverse effects.

These optimized extraction conditions derived from the green morphotype were applied to the variegated morphotype based on the following considerations: (1) both morphotypes belong to the same species and share a broadly comparable phytochemical matrix; (2) the primary objective of the comparative analysis was to evaluate differences in phenolic composition and antioxidant capacity between the two morphotypes under a standardized set of extraction conditions, rather than to independently optimize extraction for each morphotype; and (3) the limited availability of raw material from the variegated morphotype precluded the conduct of a full independent optimization experiment. Under optimized extraction conditions, both morphotypes of *A. bettzickiana* exhibited TPC values of 4.02–4.95 mg GAE/g DW and TFC values of 25.78–31.95 mg QE/g DW, while TAC of 7.04 mg C3GE/g DW was detected exclusively in the variegated morphotype. Given that the green and variegated morphotypes yielded extraction recoveries of 29.2% and 27.5%, respectively, TPCs were subsequently calculated as 14.62–16.95 mg GAE/g extract and TFCs as 88.29–116.18 mg QE/g extract. In comparison with the sole previous report on the bioactive compound contents of an ethanolic extract of *A. bettzickiana* (green) young leaves, the TPC values obtained in the present study were higher than those previously reported (TPC of 6.10 mg GAE/g extract) (Saengha et al. 2022). However, a direct comparison of TFC values with those of the aforementioned report (168.07 mg rutin equivalent (RE)/g extract) (Saengha et al. 2022) was not possible due to differences in the reference standards employed. Additional reports concerning unspecified morphotypes indicated that: (i) an ethanolic extract of aerial parts exhibited a TPC of 7.61 mg GAE/g sample and a TFC of 70.66 mg QE/g sample (Manan et al. 2020); (ii) a methanolic extract of leaves exhibited a TPC of 63 mg GAE/g extract and a TFC of 38.98 mg QE/g extract (Vidhya et al. 2015); and (iii) an 80% (*v/v*) aqueous methanolic extract of fresh leaves exhibited a TPC of 308.526 mg GAE/100 g fresh weight (Petrus et al. 2014). Transcriptomic and metabolomic analyses have indicated that the green morphotype of *A. bettzickiana* exhibits markedly low expression of key genes involved in the anthocyanin biosynthesis pathway, resulting in low anthocyanin accumulation (Li et al. 2022). This morphotype nevertheless possesses mature chloroplasts, conferring its characteristic green coloration. In contrast, the variegated morphotype exhibits elevated anthocyanin and chlorophyll contents, producing the red‐and‐green variegated phenotype and reflecting a functional balance between pigmentation and photosynthetic efficiency (Li et al. 2022).

Comparative analysis between the two morphotypes revealed that the green morphotype of *A. bettzickiana* exhibited higher TPC as determined by the Folin–Ciocalteu spectrophotometric assay; however, phenolic profiling by LC‐ESI‐MS/MS indicated a greater abundance of individual phenolic compounds in the variegated morphotype. Additionally, the variegated morphotype demonstrated higher TFC, and TAC was detected exclusively in this morphotype. The observed discrepancy in phenolic content between the two morphotypes may be attributable to several factors. First, quantification by LC‐ESI‐MS/MS is inherently restricted to compounds for which authenticated reference standards are available; phenolic compounds lacking corresponding standards would therefore be excluded from quantitative analysis, potentially leading to an underestimation of the true phenolic abundance in either morphotype. This limitation is further supported by a previous non‐targeted metabolite profiling study employing LC‐ESI‐MS/MS, which identified a total of 961 metabolites across both morphotypes, with flavonoids representing the most abundant chemical category at 233 metabolites, followed by phenolic acids and lipids at 124 and 123 metabolites, respectively (Li et al. 2022). Second, the Folin–Ciocalteu reagent is not exclusively selective for phenolic compounds and is susceptible to interference from a range of co‐occurring reducing substances, including proteins, organic acids, reducing sugars, and sulfur‐containing compounds, which may lead to overestimation of TPC, particularly in the green morphotype if these non‐phenolic reducing substances are present at higher concentrations. Several of these non‐phenolic reducing substances are plausibly more abundant in the green morphotype on physiological grounds. Because the green morphotype retains fully chlorophyllous, photosynthetically active leaf tissue, whereas the variegated morphotype diverts a substantial proportion of its leaf area to anthocyanin‐pigmented, less photosynthetically active tissue (Li et al. 2022), the green morphotype would be expected to accumulate higher levels of ascorbic acid, soluble sugars, and photosynthesis‐associated proteins. Ascorbate biosynthesis and accumulation are closely linked to chloroplast activity and light‐driven photosynthetic electron transport, such that fully green, high‐chlorophyll leaf tissue typically contains substantially larger ascorbate pools than tissue with reduced photosynthetic capacity (Akram et al. 2017; Tóth 2023). Similarly, soluble sugars, as direct products of photosynthesis, and photosynthesis‐associated proteins such as Rubisco and other chlorophyll‐binding proteins would also be expected to occur at higher concentrations in the more fully photosynthetic green tissue. As ascorbic acid, reducing sugars, and proteins are all known to react with the Folin–Ciocalteu reagent, their comparatively greater abundance in the green morphotype offers a plausible, testable physiological explanation for its higher spectrophotometric TPC value despite the variegated morphotype exhibiting a greater abundance of individually quantified phenolic compounds by LC–ESI–MS/MS. Direct quantification of ascorbic acid, soluble sugar, and protein content in both morphotypes would be required to confirm this explanation and is recommended for future studies. Third, given that both morphotypes were grown under equivalent environmental conditions, the contrasting coloration likely reflects inherent genetic differences in secondary metabolite biosynthesis (Li et al. 2022). The preferential accumulation of anthocyanins in the variegated morphotype suggests divergent regulation of the phenylpropanoid and flavonoid biosynthetic pathways between the two morphotypes, which may give rise to distinct phenolic profiles not fully captured by colorimetric assays or uniformly detected across different analytical platforms. Finally, although extraction conditions were optimized using *A. bettzickiana* (green) as a model system due to the greater availability of raw material, and subsequently applied to both morphotypes, it should be noted that the optimal extraction conditions may differ between the two morphotypes. Differences in cell wall composition and vacuolar compartmentalization may differentially affect the extractability of phenolic compounds, which is further governed by compound‐specific physicochemical properties such as polarity, molecular weight, and degree of glycosylation. Consequently, the application of a single standardized extraction protocol may not equally maximize phenolic yield from both morphotypes, thereby contributing to the observed discrepancies between spectrophotometric and chromatographic analyses.

LC‐ESI‐MS/MS analysis of both morphotypes identified *p*‐coumaric acid as the predominant phenolic compound, followed by 3,4‐dihydroxybenzoic acid, while rutin was detected only in trace amounts in the green morphotype. The sole previous report on the phenolic composition of an acid hydrolysis in methanol of *A. bettzickiana* (green morphotype), analyzed by HPLC, identified ferulic acid as the predominant compound, followed by myricetin, with rutin detected in trace amounts (Saengha et al. 2022). A separate study employing gel chromatography with a Sephadex LH‐20 column reported that an 80% (*v/v*) aqueous methanolic extract of *A. bettzickiana* fresh leaves (unspecified morphotype) was dominated by 7‐*O*‐β‐D‐glucopyranosyl‐6‐*C*‐β‐D‐glucopyranosylapigenin, with additional apigenin derivatives and hydroxycinnamate subclass compounds also detected (Petrus et al. 2014). In an ethanolic extract of aerial parts of *A. bettzickiana* (unspecified morphotype), HPLC analysis identified catechin as the predominant compound, followed by gallic acid, sinapic acid, chlorogenic acid, and α‐tocopherol, with quercetin and γ‐tocopherol present in trace amounts (Manan et al. 2020). The observed variation in phenolic identification and quantification across studies is likely attributable to both extrinsic factors, including growth environment, extraction method, and analytical platform, and intrinsic factors, including morphotype, plant parts, and developmental maturity.

Numerous studies have demonstrated a strong positive correlation between phenolic content and antioxidant activity (Muflihah et al. 2021; Sahasakul et al. 2025). Consistent with the higher TPC observed in *A. bettzickiana* (green), this morphotype demonstrated superior antioxidant activity as determined by both the SET‐based FRAP assay and the HAT‐based ORAC assay relative to the variegated morphotype. In contrast, no significant difference in DPPH radical scavenging activity was detected between the two morphotypes. Notably, although the variegated morphotype exhibited higher TFC and was the only morphotype in which TAC was detected, it nonetheless showed lower FRAP and ORAC activity than the green morphotype. This apparent discrepancy suggests that antioxidant capacity in this species is not simply a function of total flavonoid or anthocyanin quantity, but instead depends on the specific structural features of the individual compounds present, such as the degree of hydroxylation, glycosylation, and conjugation, which govern redox activity independently of total colorimetric TFC or TAC content. Because the colorimetric TFC and TAC assays quantify broad compound classes without distinguishing individual antioxidant potency, a morphotype with higher TFC or TAC does not necessarily possess proportionally higher antioxidant capacity. The predominant phenolic compound identified in both morphotypes, *p*‐coumaric acid, is known to exert antioxidant activity through multiple mechanisms, encompassing both HAT and radical adduct formation (RAF) pathways (Berton et al. 2025). Similarly, 3,4‐dihydroxybenzoic acid, the secondary phenolic compound detected in both morphotypes, is primarily recognized as a HAT‐based antioxidant owing to its *ortho*‐dihydroxy (catechol) functionality, which facilitates efficient hydrogen atom donation to neutralize free radicals (Milenković et al. 2017). Given that both morphotypes contained these HAT‐active compounds as their predominant phenolics, any quantitative differences in their concentrations between morphotypes would be expected to be more readily detected by the ORAC assay, which operates exclusively through the HAT mechanism, than by the DPPH radical scavenging assay, which is mechanistically broader and less selective for HAT‐based antioxidant activity. This mechanistic alignment between the predominant phenolic compounds and the ORAC assay may therefore account for the more pronounced between‐morphotype differences observed in ORAC values relative to DPPH radical scavenging activity. Furthermore, the DPPH radical scavenging assay has recognized inherent limitations, being considered more appropriate for evaluating hydrophobic antioxidants (Echegaray et al. 2021). This hydrophobic bias may render the DPPH radical scavenging assay less sensitive to the predominantly hydrophilic phenolic compounds present in *A. bettzickiana* extracts, thereby further attenuating the detectable difference in radical scavenging activity between the two morphotypes.

From a practical standpoint, the phenolic, flavonoid, and anthocyanin profiles characterized in this study support the potential valorization of both *A. bettzickiana* morphotypes as functional food ingredients. The higher TPC, FRAP, and ORAC activities of the green morphotype align with its established culinary use and suggest suitability as a source of antioxidant‐rich extracts or dried leaf powder for food fortification. Notably, the variegated morphotype, despite its limited culinary utilization to date, exhibited comparable or superior TFC and TAC, together with exclusive detection of anthocyanins, properties that are increasingly sought after in the development of natural colorants and anthocyanin‐based nutraceuticals. Given the reported associations between anthocyanin and flavonoid intake and reduced cardiovascular and metabolic disease risk (Pan et al. 2025; Parmenter et al. 2020), these findings support incorporating the variegated morphotype into functional food formulations, extending its utility beyond ornamental use. Further work evaluating the bioavailability, stability, and safety of these extracts would be required to translate these findings into commercial functional food or nutraceutical products.

## 5 Conclusion

Beyond the limited phytochemical and bioactivity data on *A. bettzickiana* , morphological diversity has contributed to significant inconsistencies in reported profiles. Since no validated extraction protocol exists for this species, a BBD‐based RSM approach was applied, and the optimized conditions were 90% (*v/v*) aqueous ethanol, 50°C, 2 h incubation, and 10 mg/mL extract concentration. Under these conditions, the green morphotype exhibited significantly higher TPC, FRAP, and ORAC activities, while the variegated morphotype showed greater TFC and TAC. Insignificant differences in DPPH radical scavenging activities were observed between morphotypes. LC‐ESI‐MS/MS analysis detected *p*‐coumaric acid as the predominant phenolic compound, followed by 3,4‐dihydroxybenzoic acid, in both morphotypes. This study provides the first systematic comparison of bioactive compounds and antioxidant potential between the green and variegated morphotypes of *A. bettzickiana* under optimized extraction conditions, establishing a reproducible methodological foundation for future investigations. Notably, the variegated morphotype demonstrated comparable or superior flavonoid and anthocyanin contents relative to the edible green morphotype, supporting its potential as a functional food or nutraceutical source. However, several limitations warrant acknowledgment. Extraction optimization was conducted exclusively using the green morphotype as a model system, and the resulting protocol may not equally maximize bioactive compound recovery from the variegated morphotype, owing to potential differences in cell wall composition, pigment profile, and the physicochemical properties of the constituent compounds. It is therefore recommended that a dedicated and independent extraction optimization experiment be conducted for *A. bettzickiana* (variegated) in future studies to establish morphotype‐specific extraction conditions and to ensure the reliability of subsequent bioactivity assessments. The preliminary extraction parameters were optimized using a one‐at‐a‐time (OFAT) approach, which does not account for potential interaction effects among ethanol concentration, shaking temperature, incubation time, and extract concentration. While the subsequent Box–Behnken design captured interactions among these four variables at the levels tested, future studies could employ a Plackett–Burman screening design to more rigorously evaluate variable significance and interactions prior to range selection, particularly when a larger number of candidate extraction variables is under consideration. Besides, LC–ESI–MS/MS quantification was restricted to compounds for which authenticated reference standards were available, which may have resulted in an underestimation of the full phenolic diversity present in both morphotypes. Additionally, plant material for both morphotypes was collected from a single site and harvest time, and the observed phytochemical differences should therefore be interpreted as morphotype‐associated differences under these specific growing conditions rather than as evidence that cultivar identity alone is the determining factor. Future studies incorporating multiple harvest times, locations, and/or controlled growth conditions are recommended to clarify the relative contributions of genetic (cultivar) versus environmental factors to the phytochemical profiles of *A. bettzickiana* . Future investigations employing non‐targeted metabolomic approaches are therefore recommended to provide a more comprehensive characterization of the phenolic profiles of both *A. bettzickiana* morphotypes.

## Author Contributions

**Sirinapa Thangsiri:** writing – original draft, visualization, validation, investigation, data curation, formal analysis, funding acquisition. **Amornrat Aursalung:** investigation, data curation, writing – original draft. **Uthaiwan Suttisansanee:** conceptualization, methodology, validation, formal analysis, supervision, funding acquisition, data curation, visualization, project administration, resources, writing – original draft, writing – review and editing. **Woorawee Inthachat:** data curation, writing – original draft, visualization. **Yuraporn Sahasakul:** validation, formal analysis, data curation, visualization, writing – original draft. **Piya Temviriyanukul:** conceptualization, visualization, data curation, writing – original draft, writing – review and editing.

## Funding

This research paper was supported by Institute of Nutrition, Mahidol University to S.T. and U.S. (INMU01/2568). The article processing charge (APC) was paid by Mahidol University.

## Conflicts of Interest

The authors declare no conflicts of interest.

## Supporting information


**Table S1:** Colorimetric parameters and moisture contents of powdered *Alternanthera bettzickiana* (green) and *Alternanthera bettzickiana* (variegated).
**Table S2:** Fragment ions and radio frequency (RF) of 26 authentic standards of phenolics using liquid chromatography‐electrospray ionization‐tandem mass spectrometry (LC‐ESI‐MS/MS) in selective reaction monitoring (SRM) mode*.
**Table S3:** The validation parameters of 26 authentic standards of phenolics using liquid chromatography‐electrospray ionization‐tandem mass spectrometry (LC‐ESI‐MS/MS) in selective reaction monitoring (SRM) mode*.
**Table S4:** Assumption testing (normality and variance homogeneity) prior to unpaired *t*‐tests comparing *Alternanthera bettzickiana* (green) and *A. bettzickiana* (variegated).
**Table S5:** A liquid chromatography‐electrospray ionization‐tandem mass spectrometry (LC‐ESI‐MS/MS) integration result of aqueous ethanolic extract of *Alternanthera bettzickiana* (green).
**Table S6:** A liquid chromatography‐electrospray ionization‐tandem mass spectrometry (LC‐ESI‐MS/MS) integration result of aqueous ethanolic extract of *Alternanthera bettzickiana* (variegated).

## Data Availability

The original contributions presented in the study are included in the article/Supporting Information—S1 further inquiries can be directed to the corresponding author/s.
