# Supplementary material for: Comparative Phytochemical Profiles and Antioxidant Potential of Green and Variegated Morphotypes of Alternanthera bettzickiana Under Optimized Extraction Conditions
Source: Food Sci Nutr. 2026 Aug 11;14(8):e72237. doi: 10.1002/fsn3.72237 (PMC13462314; doi:10.1002/fsn3.72237)
Supplement: Supplementary file 1 [file FSN3-14-e72237-s001.docx]

**Supplementary Material**

**Comparative Phytochemical Profiles and Antioxidant Potential of Green and Variegated Morphotypes of Alternanthera bettzickiana Under Optimized Extraction Conditions**

**Table S1.** Colorimetric parameters and moisture contents of powdered Alternanthera bettzickiana (green) and Alternanthera bettzickiana (variegated).

| **Analyses** | | | **Samples** | |
| --- | --- | --- | --- | --- |
|  |  |  | **A. bettzickiana (green)** | **A. bettzickiana (variegated)** |
| Colorimetric parameters ^1^ | Fresh samples | L* | 33.64 ± 0.21 | 34.08 ± 0.54 |
|  |  | a* | -9.01 ± 0.09 | -6.63 ± 0.18 |
|  |  | b* | 21.80 ± 0.29 | 18.79 ± 0.05 |
|  | Powdery samples | L* | 41.72 ± 0.05 | 40.23 ± 0.13 |
|  |  | a* | -5.50 ± 0.04 | -3.39 ± 0.04 |
|  |  | b* | 18.96 ± 0.03 | 16.68 ± 0.01 |
| Moisture contents (%) | | | 9.08 ± 0.66 | 6.70 ± 0.47 |

All values are expressed as mean ± standard deviation (SD) of triplicate measurements conducted on three independent samples (*n* = 3). ^1^ Color values are reported in CIELAB units, comprising lightness (L*), the red–green chromatic axis (a*), and the yellow–blue chromatic axis (b*).

**Table S2.** Fragment ions and **radio frequency (RF)** of twenty-six authentic standards of phenolics using liquid chromatography-electrospray ionization-tandem mass spectrometry (LC-ESI-MS/MS) in selective reaction monitoring (SRM) mode*.

| **Compounds** | **Standards** | **Ion mass** | **Parent ions (*m/z*)** | **SRM transitions (*m/z*) and collision energy (*V*)** | **RF lens (V)** |
| --- | --- | --- | --- | --- | --- |
| 1 | Epigallocatechin gallate | [M−H] | 457.175 | 305.155 (16.84 V), 168.97 (17.59 V), 125.042 (40.30 V) | 204 |
| 2 | Gallic acid | [M−H] | 169.05 | 124.988 (14.56 V), 96.917 (18.77 V), 79.185 (22.94 V) | 147 |
| 3 | 3,4-Dihydroxybenzoic acid | [M−H] | 152.95 | 109.113 (14.35 V), 81.042 (20.50 V), 91.042 (24.59 V) | 128 |
| 4 | Chlorogenic acid | [M−H] | 353.075 | 179.042 (14.06 V), 191.000 (16.54 V), 85.095 (39.96 V) | 148 |
| 5 | Mangiferin | [M+H] | 423.10 | 273.054 (23.58V), 303.113 (17.76V),327.071(16.88V) | 108 |
| 6 | 4-Hydroxybenic acid | [M−H] | 137.05 | 92.970 (14.86 V), 65.000 (29.39 V), 75.000 (31.96 V) | 110 |
| 7 | Caffeic acid | [M−H] | 179.038 | 135.054 (15.07 V), 107.071 (22.57 V), 85.042 (31.96 V) | 151 |
| 8 | Syringic acid | [M−H] | 197.138 | 182.185 (13.72 V), 167.113 (19.24 V), 123.095 (22.31 V) | 130 |
| 9 | Vanillic acid | [M−H] | 167.000 | 123.042 (11.66 V), 151.97 (14.59 V), 108.042 (18.65 V) | 114 |
| 10 | *p*-Coumaric acid | [M+H] | 165.05 | 147.054 (11.70 V), 119.113 (19.36 V), 91.125 (25.89 V) | 90 |
| 11 | Rutin | [M+H] | 611.20 | 303.13 (20.80), 465.20 (12.71V) | 198 |
| 12 | Sinapic acid | [M−H] | 223.25 | 208.125 (13.51 V), 164.024 (15.78 V), 192.970 (22.65 V) | 141 |
| 13 | Ferulic acid | [M−H] | 192.95 | 149.125 (11.28 V), 177.970 (13.05 V), 134.042 (16.50 V) | 124 |
| 14 | Hesperidin | [M−H] | 609.30 | 301.179 (24.50 V), 325.179 (27.83 V), 286.125 (41.60 V) | 299 |
| 15 | Myricetin | [M−H] | 317.088 | 178.970 (19.53 V), 150.988 (24.50 V), 137.113 (26.86 V) | 245 |
| 16 | Rosmarinic acid | [M−H] | 359.20 | 197.000 (15.70 V), 161.113 (17.38 V), 133.054 (37.81 V) | 175 |
| 17 | Fisetin | [M+H] | 287.05 | 213.054(27.96V), 137.042 (30.86V), 241.125(25.09V) | 244 |
| 18 | Luteolin | [M−H] | 285.138 | 197.000 (15.70 V), 161.113 (17.38 V), 133.054 (37.81 V) | 241 |
| 19 | Quercetin | [M−H] | 301.200 | 178.976 (18.18 V), 273.125 (19.45 V), 151.042 (21.39 V) | 237 |
| 20 | Cinnamic acid | [M−H] | 147.00 | 103.00 (11.23V), 77.083 (23.07) | 107 |
| 21 | Apigenin | [M−H] | 269.075 | 116.863 (34.28 V), 149.071 (25.13 V), 151.131 (25.05 V) | 244 |
| 22 | Genistein | [M−H] | 269.138 | 224.054 (25.60 V), 159.054 (29.26 V), 132.929 (30.95 V) | 239 |
| 23 | Naringenin | [M+H] | 272.938 | 146.97 (21.01 V), 153.054 (24.42 V), 119.000 (31.28 V) | 160 |
| 24 | Kaempferol | [M−H] | 285.150 | 184.911 (25.85 V), 239.113 (27.03 V), 186.988 (28.17 V) | 260 |
| 25 | Isorhamnetin | [M−H] | 315.088 | 300.000 (21.30 V), 150.970 (29.14 V), 271.054 (30.57 V) | 233 |
| 26 | Galangin | [M+H] | 271.088 | 165.042 (28.80 V), 197.125 (31.75 V), 153.113 (32.42 V) | 248 |

^*^These data are from our previous study (Sirichai et al., 2022).

**Table S3.** The validation parameters of twenty-six authentic standards of phenolics using liquid chromatography-electrospray ionization-tandem mass spectrometry (LC-ESI-MS/MS) in selective reaction monitoring (SRM) mode^*^.

| **Compounds** | **Retention time (min)** | **Standards** | **Linear range (µg/mL)** | **Linear regression equation** | **Correlation coefficient (R^2^)** | **LOD (µg/mL)** | **LOQ (µg/mL)** | **%RSD**  **(Inter-day)** | **%Recovery** | | |
| --- | --- | --- | --- | --- | --- | --- | --- | --- | --- | --- | --- |
|  |  |  |  |  |  |  |  |  | **Low level**  **(µg/mL)** | **Medium level**  **(µg/mL)** | **High level**  **(µg/mL)** |
| 1 | 0.44 | Epigallocatechin gallate | 0.125−40 | y = 8533x + 1053.4 | 0.9985 | 0.067 | 0.230 | 0.023 | 91.84 | 85.36 | 91.37 |
| 2 | 0.564 | Gallic acid | 0.195−25 | y = 3323.1x − 2100.4 | 0.9984 | 0.04 | 0.14 | 0.01 | 113.05 | 118.57 | 109.12 |
| 3 | 0.803 | 3,4-Dihydroxybenzoic acid | 0.195−25 | y = 11490x − 10877 | 0.9935 | 0.010 | 0.034 | 0.003 | 90.59 | 85.75 | 89.75 |
| 4 | 0.922 | Chlorogenic acid | 0.3125−40 | y = 8377.5x − 3623.5 | 0.9934 | 0.017 | 0.055 | 0.006 | 91.94 | 87.50 | 95.02 |
| 5 | 1.116 | Mangiferin | 0.125−10 | y = 7516.2x + 195.48 | 0.9985 | 0.040 | 0.130 | 3.700 | 102.20 | 91.14 | 91.57 |
| 6 | 1.16 | 4-Hydroxybenic acid | 0.3125−40 | y = 2482.6x− 3998.4 | 0.9917 | 0.027 | 0.090 | 0.009 | 109.67 | 103.60 | 101.28 |
| 7 | 1.40 | Caffeic acid | 0.3125−40 | y = 12328x−19725 | 0.9918 | 0.010 | 0.035 | 0.003 | 105.36 | 93.98 | 87.41 |
| 8 | 1.539 | Syringic acid | 3.125−100 | y = 68.091x + 230.43 | 0.9955 | 0.582 | 1.939 | 0.194 | 116.35 | 97.42 | 94.91 |
| 9 | 1.63 | Vanillic acid | 2.5−100 | y = 213.67x − 975.72 | 0.9900 | 0.15 | 0.48 | 0.05 | 99.86 | 101.76 | 100.12 |
| 10 | 2.452 | *p*-Coumaric acid | 0.3125−40 | y = 8532.4x−13559 | 0.9910 | 0.013 | 0.042 | 0.004 | 88.22 | 81.36 | 98.05 |
| 11 | 2.737 | Rutin | 0.009−1.25 | y = 49729x − 33.064 | 0.9999 | 0.001 | 0.005 | 0.0005 | 94.63 | 114.00 | 108.73 |
| 12 | 2.772 | Sinapic acid | 0.39−25 | y = 1592.6x − 832.22 | 0.9977 | 0.026 | 0.086 | 0.009 | 81.34 | 92.16 | 84.22 |
| 13 | 2.851 | Ferulic acid | 1.56−100 | y = 559.03x − 1819.2 | 0.9947 | 0.155 | 0.518 | 0.052 | 91.51 | 89.24 | 93.10 |
| 14 | 3.41 | Hesperidin | 0.25−40 | y = 838.63x − 242.2 | 0.9986 | 0.07 | 0.22 | 0.02 | 100.43 | 104.06 | 108.60 |
| 15 | 3.431 | Myricetin | 1.25−40 | y = 303.47x − 601.81 | 0.9976 | 0.261 | 0.871 | 0.087 | 113.07 | 81.77 | 91.12 |
| 16 | 3.528 | Rosmarinic acid | 0.3125−40 | y = 4322.4x-3744.1 | 0.9956 | 0.07 | 0.25 | 0.02 | 92.45 | 106.35 | 99.62 |
| 17 | 3.620 | Fisetin | 0.25−10 | y = 7468.4x − 701.27 | 0.9979 | 0.150 | 0.490 | 6.900 | 98.01 | 108.94 | 117.51 |
| 18 | 4.158 | Luteolin | 0.195−12.5 | y = 8381.9x − 5000.7 | 0.9945 | 0.015 | 0.050 | 0.0005 | 84.21 | 96.21 | 107.09 |
| 19 | 4.185 | Quercetin | 0.05−12.5 | y = 2934x + 917.17 | 0.9937 | 0.05 | 0.18 | 0.02 | 83.36 | 115.06 | 95.74 |
| 20 | 4.522 | Cinnamic acid | 0.039−10 | y = 6631.9x − 866.59 | 0.9964 | 0.049 | 0.163 | 0.016 | 101.94 | 98.84 | 95.85 |
| 21 | 4.689 | Apigenin | 0.34−11 | y = 1790.7x − 287.7 | 0.9997 | 0.127 | 0.424 | 0.042 | 88.84 | 106.89 | 114.79 |
| 22 | 4.693 | Genistein | 0.625−40 | y = 1247.2x − 1747.1 | 0.9977 | 0.049 | 0.163 | 0.016 | 95.33 | 101.49 | 11633 |
| 23 | 4.705 | Naringenin | 0.0008−5 | y = 16755x + 443.03 | 0.9932 | 0.003 | 0.011 | 0.001 | 117.92 | 96.26 | 111.08 |
| 24 | 4.79 | Kaempferol | 0.25−10 | y = 1006.8x − 346.28 | 0.9905 | 0.122 | 0.406 | 0.041 | 92.35 | 107.69 | 102.17 |
| 25 | 4.878 | Isorhamnetin | 0.0098−2.5 | y = 12698x + 586.16 | 0.9945 | 0.016 | 0.052 | 0.005 | 113.57 | 105.88 | 111.14 |
| 26 | 6.146 | Galangin | 0.3125−40 | y = 5012.1x − 9354.7 | 0.9879 | 0.010 | 0.035 | 0.003 | 84.01 | 112.92 | 115.80 |

^*^These data are from our previous study (Sirichai et al., 2022). LOD: limit of detection; LOQ: limit of quantification; RSD: relative standard deviation.

**Table S4**. Assumption testing (normality and variance homogeneity) prior to unpaired *t*-tests comparing *Alternanthera bettzickiana* (green) and *A. bettzickiana* (variegated).

| **Parameter** | **Shapiro–Wilk (green)** | **Shapiro–Wilk (variegated)** | **Levene's test (F, *p*)** | **Variance equality** | **Test applied** | **t-statistic** | **df** | ***p*-value** |
| --- | --- | --- | --- | --- | --- | --- | --- | --- |
| 3,4-Dihydroxybenzoic acid (μg/g extract) ^*^ | 1.000 (*p* = 0.987) | 0.999 (*p* = 0.945) | 1.965 (*p* = 0.234) | Equal | Student's t-test | -2.533 | 4 | 0.064 (ns) |
| *p*-Coumaric acid (μg/g extract) ^*^ | 1.000 (*p* = 0.995) | 1.000 (*p* = 0.973) | 0.219 (*p* = 0.664) | Equal | Student's t-test | -3.955 | 4 | 0.017 |
| Rutin (μg/g extract) ^*, †^ | – | – | – | – | Not testable† | – | – | – |
| TAC (mg C3GE/g DW) ^‡^ | – | – | – | – | Not testable‡ | – | – | – |
| TPC (mg GAE/g DW) | 0.985 (*p* = 0.769) | 0.934 (*p* = 0.503) | 0.803 (*p* = 0.421) | Equal | Student's t-test | 6.801 | 4 | 0.002 |
| TFC (mg QE/g DW) | 0.904 (*p* = 0.399) | 0.791 (*p* = 0.093) | 0.998 (*p* = 0.374) | Equal | Student's t-test | -3.991 | 4 | 0.016 |
| DPPH (μmol TE/g DW) | 0.950 (*p* = 0.567) | 0.992 (*p* = 0.828) | 0.192 (*p* = 0.684) | Equal | Student's t-test | 0.893 | 4 | 0.422 (ns) |
| FRAP (μmol TE/g DW) | 0.959 (*p* = 0.610) | 0.837 (*p* = 0.206) | 1.253 (*p* = 0.326) | Equal | Student's t-test | 3.036 | 4 | 0.039 |
| ORAC (μmol TE/g DW) | 0.928 (*p* = 0.480) | 0.972 (*p* = 0.678) | 0.367 (*p* = 0.577) | Equal | Student's t-test | 6.166 | 4 | 0.004 |

Normality was assessed using the Shapiro–Wilk test and variance homogeneity using Levene's test, separately for each species group. Two levels of replication were used in this study: biological replicates (independent sample sets, *n* = 3) and technical replicates (triplicate measurements performed on each biological replicate). For TPC, TFC, and antioxidant activities measured by DPPH radical scavenging, FRAP, and ORAC assays, the mean of the three technical replicates was calculated first for each biological replicate, and all statistics reported here (Shapiro–Wilk, Levene's, and *t*-test) are based on the resulting three biological replicate means per species (*n* = 3), not on the nine individual technical measurements, to avoid pseudoreplication. df: degrees of freedom; ns: not significant (*p* > 0.05). At *n* = 3, the Shapiro–Wilk test has very low power and high *p*-values should not be interpreted as strong evidence of normality; all results in this table should be interpreted with this caveat in mind. All statistical comparisons in the manuscript were evaluated at *p* < 0.05.

* LC-ESI-MS/MS compounds had no technical replication (one injection per biological replicate; *n* = 3 per species). Where Levene's test indicated unequal variances (*p* < 0.05), Welch's correction would be applied; here all parameters showed equal variances, so Student's unpaired *t*-test was used throughout.

^†^Rutin was not detected (ND) in *A. bettzickiana* (variegated), so no comparison was possible.

^‡^For TAC, *A. bettzickiana* (green) yielded negative computed values, reflecting negligible/non-detectable anthocyanin content (ND) rather than genuine measurements; TAC could therefore not be statistically compared between species, and only *A. bettzickiana* (variegated) (7.04 ± 0.63 mg C3GE/g DW) is reported.

**Table S5**. A liquid chromatography-electrospray ionization-tandem mass spectrometry (LC-ESI-MS/MS) integration result of aqueous ethanolic extract of Alternanthera bettzickiana (green).

| No | Peak Name | Retention time (min) | Quantitation ion | Area (counts*min) | Height (counts) | Overall ion ratio Confirmation |
| --- | --- | --- | --- | --- | --- | --- |
| 1 | ECG | n.a. | 168.97 | n.a. | n.a. | Not confirmed |
| 2 | Gallic acid | n.a. | 124.99 | n.a. | n.a. | Not confirmed |
| 3 | Chlorogenic acid | 0.660 | 191.00 | 9 | 215 | Not confirmed |
| 4 | 3,4-Dihydroxybenzoic acid | 0.772 | 109.11 | 3917 | 57758 | confirmed |
| 5 | Mangiferin | 1.158 | 273.05 | 18 | 1553 | Not confirmed |
| 6 | 4-Hydroxybenzoic acid | 1.249 | 92.97 | 649 | 5713 | Not confirmed |
| 7 | Caffeic acid | 1.510 | 135.00 | 368 | 3903 | Not confirmed |
| 8 | Syringic acid | 1.557 | 182.18 | 15 | 165 | Not confirmed |
| 9 | Vanillic acid | n.a. | 151.97 | n.a. | n.a. | Not confirmed |
| 10 | *p*-Coumaric acid | 2.537 | 147.05 | 2751 | 41477 | Confirmed |
| 11 | Rutin | 2.704 | 303.13 | 912 | 25137 | Confirmed |
| 12 | Sinapic acid | n.a. | 208.13 | n.a. | n.a. | Not confirmed |
| 13 | Ferulic acid | 2.880 | 134.04 | 49 | 1338 | Not confirmed |
| 14 | Hesperidin | 3.302 | 301.18 | 9 | 502 | Not confirmed |
| 15 | Myricetin | n.a. | 150.99 | n.a. | n.a. | Not confirmed |
| 16 | Fisetin | 3.559 | 213.05 | 29 | 1812 | Not confirmed |
| 17 | Rosmarinic acid | 3.571 | 161.11 | 10 | 678 | Not confirmed |
| 18 | Luteolin | 4.142 | 133.07 | 11 | 728 | Not confirmed |
| 19 | Quercetin | 4.223 | 151.04 | 6 | 208 | Not confirmed |
| 20 | Cinnamic acid | 4.486 | 103.00 | 6 | 758 | Not confirmed |
| 21 | Genistein | n.a. | 132.93 | n.a. | n.a. | Not confirmed |
| 22 | Naringenin | 4.702 | 153.05 | 63 | 1429 | Not confirmed |
| 23 | Apigenin | 4.705 | 116.86 | 22 | 511 | Not confirmed |
| 24 | Kaempferol | 4.785 | 239.11 | 50 | 1196 | Not confirmed |
| 25 | Isorhamnetin | 4.894 | 300.00 | 113 | 2967 | Not confirmed |
| 26 | Galangin | 6.172 | 153.11 | 1 | 65 | Not confirmed |

n.a.: Not available.

**Table S6**. A liquid chromatography-electrospray ionization-tandem mass spectrometry (LC-ESI-MS/MS) integration result of aqueous ethanolic extract of Alternanthera bettzickiana (variegated).

| No | Peak Name | Retention time (min) | Quantitation ion | Area (counts*min) | Height (counts) | Overall ion ratio Confirmation |
| --- | --- | --- | --- | --- | --- | --- |
| 1 | ECG | n.a. | 168.97 | n.a. | n.a. | Not confirmed |
| 2 | Gallic acid | 0.512 | 124.99 | 10 | 620 | Not confirmed |
| 3 | Chlorogenic acid | 0.689 | 191.00 | 80 | 788 | Not confirmed |
| 4 | 3,4-Dihydroxybenzoic acid | 0.772 | 109.11 | 5468 | 78057 | Confirmed |
| 5 | Mangiferin | 1.187 | 273.05 | 9 | 644 | Not confirmed |
| 6 | 4-Hydroxybenzoic acid | 1.239 | 92.97 | 534 | 4632 | Not confirmed |
| 7 | Caffeic acid | 1.523 | 135.00 | 614 | 5848 | Not confirmed |
| 8 | Vanillic acid | 1.542 | 151.97 | 4 | 68 | Not confirmed |
| 9 | Syringic acid | 1.594 | 182.18 | 4 | 53 | Not confirmed |
| 10 | *p*-Coumaric acid | 2.532 | 147.05 | 3044 | 52662 | Confirmed |
| 11 | Rutin | 2.689 | 303.13 | 243 | 3718 | Not confirmed |
| 12 | Sinapic acid | n.a. | 208.13 | n.a. | n.a. | Not confirmed |
| 13 | Ferulic acid | 2.875 | 134.04 | 25 | 1377 | Not confirmed |
| 14 | Hesperidin | 3.330 | 301.18 | 11 | 665 | Not confirmed |
| 15 | Myricetin | n.a. | 150.99 | n.a. | n.a. | Not confirmed |
| 16 | Rosmarinic acid | 3.533 | 161.11 | 2 | 102 | Not confirmed |
| 17 | Fisetin | 3.544 | 213.05 | 22 | 1196 | Not confirmed |
| 18 | Luteolin | 4.184 | 133.07 | 5 | 309 | Not confirmed |
| 19 | Quercetin | 4.236 | 151.04 | 2 | 104 | Not confirmed |
| 20 | Cinnamic acid | 4.532 | 103.00 | 7 | 824 | Not confirmed |
| 21 | Genistein | n.a. | 132.93 | n.a. | n.a. | Not confirmed |
| 22 | Apigenin | 4.670 | 116.86 | 30 | 926 | Not confirmed |
| 23 | Naringenin | 4.725 | 153.05 | 26 | 1088 | Not confirmed |
| 24 | Kaempferol | n.a. | 239.11 | n.a. | n.a. | Not confirmed |
| 25 | Isorhamnetin | 4.893 | 300.00 | 112 | 2217 | Not confirmed |
| 26 | Galangin | 6.147 | 153.11 | 7 | 426 | Not confirmed |

n.a.: Not available.

**Reference**

Sirichai, P., Kittibunchakul, S., Thangsiri, S., On-Nom, N., Chupeerach, C., Temviriyanukul, P., Inthachat, W., Nuchuchua, O., Aursalung, A., Sahasakul, Y., Charoenkiatkul, S., Suttisansanee, U., Impact of drying processes on phenolics and in vitro health-related activities of indigenous plants in Thailand, Plants (Basel). 11(3) (2022), doi: 10.3390/plants11030294.
